# Reconstruction of the evolution of microbial defense systems

**DOI:** 10.1186/s12862-017-0942-y

**Published:** 2017-04-04

**Authors:** Pere Puigbò, Kira S. Makarova, David M. Kristensen, Yuri I. Wolf, Eugene V. Koonin

**Affiliations:** 1 National Center for Biotechnology Information, National Library of Medicine, National Institutes of Health, Bethesda, MD 20894 USA; 2 Present address: Division of Genetics and Physiology, Department of Biology, University of Turku, Turku, Finland; 3 Present address: Department of Biomedical Engineering, University of Iowa, Iowa City, IA USA

## Abstract

**Background:**

Evolution of bacterial and archaeal genomes is a highly dynamic process that involves intensive loss of genes as well as gene gain via horizontal transfer, with a lesser contribution from gene duplication. The rates of these processes can be estimated by comparing genomes that are linked by an evolutionary tree. These estimated rates of genome dynamics events substantially differ for different functional classes of genes. The genes involved in defense against viruses and other invading DNA are among those that are gained and lost at the highest rates.

**Results:**

We employed a stochastic birth-and-death model to obtain maximum likelihood estimates of the rates of gain and loss of defense genes in 35 groups of closely related bacterial genomes and one group of archaeal genomes. We find that on average, the defense genes experience 1.4 fold higher flux than the rest of microbial genes. This excessive flux of defense genes over the genomic mean is consistent across diverse microbial groups. The few exceptions include intracellular parasites with small, degraded genomes that possess few defense systems which are more stable than in other microbes. Generally, defense genes follow the previously established pattern of genome dynamics, with gene family loss being about 3 times more common than gain and an order of magnitude more common than expansion or contraction of gene families. Case by case analysis of the evolutionary dynamics of defense genes indicates frequent multiple events in the same locus and widespread involvement of mobile elements in the gain and loss of defense genes.

**Conclusions:**

Evolution of microbial defense systems is highly dynamic but, notwithstanding the host-parasite arms race, generally follows the same trends that have been established for the rest of the genes. Apart from the paucity and the low flux of defense genes in parasitic bacteria with deteriorating genomes, there is no clear connection between the evolutionary regime of defense systems and microbial life style.

**Electronic supplementary material:**

The online version of this article (doi:10.1186/s12862-017-0942-y) contains supplementary material, which is available to authorized users.

## Background

Horizontal gene transfer (HGT) that can result in genome expansion and acquisition of new functions and gene loss leading to genome reduction are recognized as key processes in the evolution of bacterial and archaeal genomes [1–3]. It has been quantitatively demonstrated that gene gain via HGT rather than intragenomic gene duplication is the principal factor of innovation in the evolution of prokaryotes [4]. Reconstructions of gene gain and loss in groups of closely related bacteria and archaea reveal genomes that are in constant flux, with high rates of gain and loss [5–7]. In many cases, several events of gene gain and loss have been estimated to occur over the time that it takes for a single nucleotide substitution to be fixed in an average gene [7]. According to these reconstructions, gene loss appears to be the most common process in microbial evolution that occurs in a roughly clock-like manner [8]. Gene gain appears to be a more episodic process that occurs at rates that on average are two to three-fold lower than the gene loss rate which might be compensated for by bursts of gene gain [8].

Nearly all genes can be transferred and lost during the evolution of bacteria and archaea but the rates of these processes markedly differ between functional classes of genes [7]. The genes involved in key processes of information transmission, in particular translation, comprise the most stable group whereas genes that encode components of the mobilome, such as transposons or prophages, predictably are lost and gained more often. On the genome dynamics scale, the mobilome is closely followed by the genes that encode various defense systems. Bacteria and archaea exist under constant pressure from parasites, such as viruses, transposons and plasmids, and have evolved elaborate, diverse, multilayer defense strategies [9–12]. The defense systems include those that provide innate immunity, such as restriction-modification (RM), toxin-antitoxin (TA), and abortive infection (AI) systems, and the CRISPR-Cas (Clustered Regularly Interspaced Repeats and CRISPR-associated proteins) systems of adaptive immunity. The incessant arms race between the parasites and the defense systems results in rapid evolution on both sides which involves sequence change as well as intense gene flux [7, 13–15].

Over the last several decades, microbial defense mechanisms have been intensely exploited as source of tools for genome editing, engineering and regulation. In the early days of genetic engineering, the key tools were RM enzymes, and the need in endonucleases with different specificities has greatly stimulated the study of the diversity of RM systems [16–18]. Later, the new era of genome editing has been ushered by the characterization of the programmable, RNA-guided endonuclease activity of CRISPR-Cas systems, particular those of Class 2 that possess a single-protein effector modules and therefore can be introduced into mammalian cells in a straightforward manner [19–21]. Notably, systematic screening of bacterial and archaeal genomes for new Class 2 CRISPR-Cas systems resulted in the discovery of several novel variants that have been immediately harnessed for applications [22–24]. Even more recently, bacterial and archaeal Argonaute proteins have been shown to represent a distinct, RNA- or DNA-guided innate immunity machinery in bacteria and archaea [25, 26] that eventually might lead to yet another generation of genome editing tools notwithstanding the controversy around the early attempts on application [27, 28]. The remarkable utility of microbial defense systems as tools for genome manipulation stems from their naturally evolved ability to recognize and cleave specific DNA or RNA sequence, which is a pre-requisite for self vs non-self discrimination. These features of defense systems undoubtedly enhance the incentive for detailed exploration of their diversity and evolution.

We undertook a detailed analysis of the gain and loss dynamics of defense systems in 36 clusters of closely related prokaryotic genome cluster (35 bacteria and 1 archaea) from an updated version of the ATGC (Alignable Tight Genome Clusters), a database of orthologous genes from closely related prokaryotic genomes and a research platform for microevolution of prokaryotes [7, 29, 30] and compared the identified trends with those for the overall genome dynamics. The results support the previous observations on the highly dynamic character of the evolution of defense systems but also show that, despite the evolution in the arms race regime, the relative contributions of different types of evolutionary events are roughly the same for defense systems as they are for the rest of microbial genes.

## Results and discussion

### Distribution of defense systems among the ATGCs

**Fig. 1:**
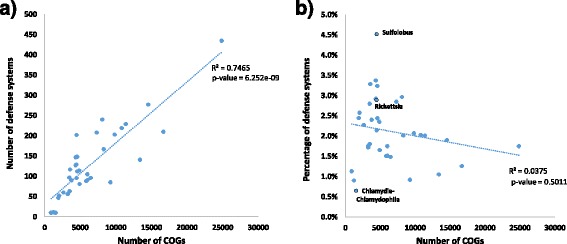
Correlation of COGs and defense systems. **a** All COGs vs. number of defense systems. **b** All COGs vs. percentage of defense systems. Each figure contains 36 dots each of which represents an ATGC. The ATGCs discussed in the text are indicated in panel **b**

In extension of the previous observations [31], the total number of defense systems (DS) genes strongly correlates with the total number of COGs in an ATGC such that about 75% of the variation is explained by the characteristic genome size (Fig. 1a). In contrast, the fraction of defense-related genes does not significantly depend on the genome size (Fig. 1b) although considerable variation is observed across the ATGCs, from <1% in *Chlamydia-Chlamydophila* to >4% in *Sulfolobus* (the only archaeon in the dataset). Not surprisingly, ATGCs with the lowest representation of defense systems include intracellular parasites *Chlamydia-Chlamydophila* (ATGC022), followed by another group of *Chlamydia* (ATGC021), and *Mycoplasma* (ATGC032), facultative, host-associated bacteria with heavily reduced genomes (Additional file 1: Table S1). *Rickettsia*, intracellular parasites with average-size genomes, also encompass an average number of defense systems (Additional file 1: Table S1). Thus, apparently, the number of defense systems is determined primarily by the size of the genome and is largely independent of the life style of the microbes.

**Fig. 2:**
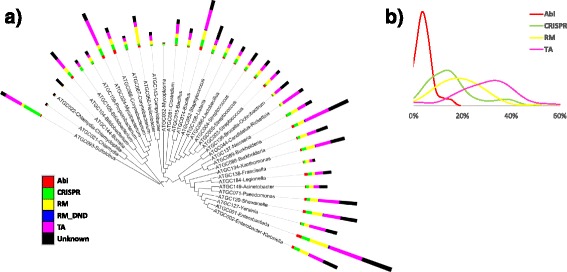
Distribution of defense systems across the species tree. **a** The tree was reconstructed using data from MicrobesOnline [56]. Length of bars is proportional to the number of defense systems. **b** Distribution of the relative abundance of defense systems in the ATGC

The distribution of the different types of defense systems is largely consistent across the ATGCs (Fig. 2 and Additional file 1: Table S1). Statistically, the fractions of each class of defense systems did not differ from the respective means for any of the ATGCs (Additional file 1: Table S2).

Overall, TA comprise the most abundant class of defense systems (on average, ~30% out of the total defense systems in an ATGC), followed by RM (~20%) and CRISPR (~15%) (here we analyzed only the type 2 TA which are more directly implicated in defense, considerably more abundant and more readily recognizable than other types of TA [32]). There is also, on average, about 30% of uncharacterized (predicted) defense systems, i.e*.* those that contain genes associated with defense but could not be classified into one of the defined categories. As shown in Fig. 2b (see also Additional file 1: Table S1), in 20 of the 36 ATGCs, TA are the most abundant defense systems. The RM systems are extremely abundant in *Helicobacter* (ATGC050), representing 54% of the DS in this ATGC, and also more abundant than other DS in *Listeria* (ATGC108), *Francisella* (ATGC138) and *Propionibacterium* (ATGC159). The CRISPR-Cas systems are the most abundant DS in *Sulfolobus* (ATGC093), followed by *Corynebacterium* (ATGC067) and *Mycoplasma* (ATGC032).

### Rates of different types of genome dynamics events in defense systems

We reconstructed the evolution of the DS in large ATGCs that include at least 10 and up to 109 genomes (Additional file 1: Table S1). In each ATGC, the gene families involved in DS were identified, and the rates of Gene Dynamics Events (GDE), including gene family gain, loss, expansion and reduction (henceforth, gain and loss refers to appearance and disappearance, respectively, of a new gene family in the given genome; expansion and reduction refer to gain and loss of a new member of a family of paralogs, respectively), were analyzed using COUNT [7, 33].

COUNT employs a phylogenetic birth-and-death model to infer three parameters: κ (rate of gene family gain), λ (individual gene duplication rate) and μ (individual gene loss rate) These parameters are used to estimate the posterior probabilities of the four types of transitions for each gene family and on all edges of the species tree, namely gain of a gene family (absence - > presence transition), gene family expansion (family of k - > k + 1, k > 0), gene family reduction (family of k - > k-1, k > 1) and loss of a gene family (presence - > absence, i.e. same as reduction but for k = 1). The posterior probabilities can be used to calculate the actual rates (effective number of events, normalized per gene) that can differ from the internal rates in the model. We chose to investigate the evolution of genomes in terms of such effective rates of the four classes of events rather than in terms of the underlying model rates because the former provide a more realistic account of the actual phyletic patterns observed by genome comparison.

The results were compared with the overall rates of the respective events within each ATGC (See Methods and Additional file 1: Figure S1 for the details on the analysis pipeline). The relative rates of gene family gain, loss, expansion and reduction among the DS are similar to those previously estimated for all genes in the ATGC [7]. The rates of gain, loss, expansion and reduction were divided by the total number of GDE and the resulting normalized relative rates were compared for DS genes vs all genes using the Chi-square test. This test yielded a *p*-value of 0.24 indicating that the DS genes do not significantly deviate from the overall pattern.

**Fig. 3:**
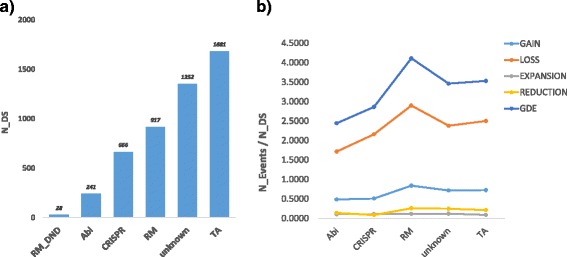
Number of genes assigned to defense systems (N_DS) and genome dynamics by type of defense system. **a** Number of defense genes (COGs) distributed by type of defense systems. **b** Number of genome dynamic events (GDE), including gain, loss, expansion and reduction, by type of defense systems

As in the case of the overall genome dynamics, gene loss is the dominant mode of evolution of defense systems, the loss rate being approximately 3-fold higher than gene gain rate and an order of magnitude higher than the gene family expansion and reduction rates (Fig. 3). Under the assumption that the genomes are at equilibrium, long term, it appears likely that the substantial excess of losses over gains is offset by sporadic gain of multiple genes [7, 8]. The proportional differences in the rates of genome dynamics events are similar for all classes of defense systems (Abi, CRISPR-Cas, RM and TA) (Fig. 3).

As expected, the number of genome dynamics events depends on the size of the ATGC. Thus, ATGC001 that consists of *Enterobacteria* (>100 genomes) encompasses the majority of the inferred events (Table 1). In order to compare genome dynamics within and across ATGCs, we normalized the estimated number of events by the number of COGs and the number of genomes in an ATGC (Additional file 1: Table S2).

**Table 1:** Genome dynamics in defense systems relative to the dynamics in all genes

ATGC	Genera	(DS/N_DS) / (ALL/N_ALL)				
		Gain	Loss	Expansion	Reduction	All GDE
ATGC149	Acinetobacter	1.73	1.31	1.09	1.89	1.39
ATGC014	Bacillus	1.06	1.18	0.71	1.16	1.13
ATGC015	Bacillus	1.17	1.68	0.62	0.73	1.47
ATGC104	Bifidobacterium	1.22	1.31	0.59	0.43	1.22
ATGC105	Bifidobacterium	1.90	1.73	0.55	1.11	1.76
ATGC144	Borrelia	0.78	1.63	1.12	1.71	1.51
ATGC136	Brucella-Ochrobactrum	1.20	0.45	0.02	0.19	0.70
ATGC088	Burkholderia	1.76	0.76	1.18	0.78	0.91
ATGC089	Burkholderia	1.34	1.24	0.24	0.51	1.20
ATGC143	Campylobacter	1.16	1.92	1.37	1.99	1.77
ATGC044	Candidatus-Rickettsia	1.01	1.44	1.22	1.85	1.34
ATGC021	Chlamydia	0.20	0.68	0.01	0.82	0.65
ATGC022	Chlamydia-Chlamydophila	0.01	0.79	1.01	0.26	0.54
ATGC081	Clostridium	1.70	1.36	1.04	2.59	1.40
ATGC067	Corynebacterium	1.46	1.17	0.70	1.08	1.19
ATGC068	Corynebacterium	1.18	1.89	1.44	1.33	1.79
ATGC002	Enterobacter-Klebsiella	1.40	1.85	1.10	3.14	1.63
ATGC001	Enterobacteria	1.18	1.98	1.61	3.51	1.88
ATGC138	Francisella	1.46	1.63	0.59	2.86	1.59
ATGC050	Helicobacter	1.22	2.90	3.15	4.97	2.63
ATGC056	Lactobacillus	1.58	1.34	0.75	1.17	1.36
ATGC184	Legionella	2.13	1.75	1.19	1.92	1.78
ATGC108	Listeria	1.23	2.69	0.42	2.29	2.29
ATGC024	Mycobacterium	0.84	1.38	0.88	0.54	1.16
ATGC032	Mycoplasma	0.34	1.51	1.29	0.19	1.32
ATGC137	Neisseria	1.35	1.18	1.43	0.76	1.22
ATGC159	Propionibacterium	1.86	0.72	1.05	0.17	0.93
ATGC071	Pseudomonas	1.54	1.45	0.69	0.96	1.42
ATGC120	Shewanella	2.87	0.99	1.31	2.52	1.34
ATGC052	Staphylococcus	1.06	1.22	0.73	1.65	1.21
ATGC003	Streptococcus	1.11	1.02	2.03	0.68	1.05
ATGC004	Streptococcus	1.29	1.45	1.48	1.57	1.42
ATGC005	Streptococcus	1.36	1.64	0.69	1.54	1.49
ATGC093	Sulfolobus	0.96	1.66	1.55	1.62	1.55
ATGC134	Xanthomonas	1.11	2.19	1.43	3.01	1.71
ATGC127	Yersinia	1.29	1.38	1.20	1.21	1.34

The number of events per COG per genome ranged between approximately 0.1 and 0.4 (Additional file 1: Table S3). The median value is 0.16, and only 4 ATGCs show values greater than 0.3 (ATGC068-*Corynebacterim* [CRISPR and TA], ATGC120-*Shewanella* [Abi], ATGC149-*Acinetobacter* [TA] and ATGC184 - *Legionella* [Abi]). There were no significant differences in genome dynamics rates between different microbial life styles (free-living vs facultative host-associated vs parasites) or across the major bacterial taxa (Additional file 1: Tables S4 and S5).

### High flux vs low flux in defense systems

**Fig. 4:**
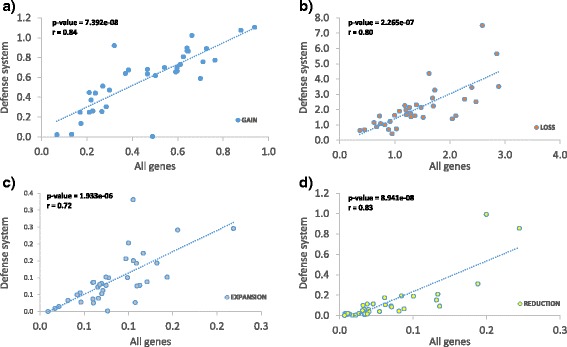
Correlation of genome dynamics events normalized by number of COGs of all genes vs. defense systems. **a** Gain. **b** Loss. **c** Expansion. **d** Reduction

Rates of gain, loss, expansion and reduction per COG in defense systems strongly correlate with the rates among all genes (Fig. 4). To identify genomes with high and low relative rates of genome dynamics in defense systems, the results were normalized by the overall rates of genome dynamics events in all genes. There are 9 ATGCs with high flux (top quartile), 9 ATGCs with low flux (bottom quartile) and 18 ATGCs with average flux of defense systems (Table 1 and Additional file 1: Table S6). *Helicobacter* and *Listeria* have the highest gene flux, whereas *Chlamydia* and *Brucella* show the lowest flux (Additional file 1: Table S6). The majority of the ATGCs with a high DS gene flux are compressing, i.e. appear to be shedding defense systems (*Enterobacteria*, in ATGC002 as well as Streptococcus, *Listeria*, *Xanthomonas* and *Campylobacter*) although two ATGCs (*Helicobacter* and *Legionella*) show high rates of both gene gain and loss. Moreover, *Helicobacter* presents the highest rates of gain, loss, expansion and reduction (Additional file 1: Table S3). In contrast, ATGCs with low flux rate tend to maintain a balanced gain/loss rate in 6 ATGCs or to slowly expand their defense systems (ATGC003-*Streptococcus*, ATGC088-*Burkolderia* and *Propionibacterium*). The same trend is observed in ATGCs with average flux: 14 ATGCs have balanced gain-loss rates, two tend to gain DS genes (*Shewanella* and *Acinetobacter*) and two tend to lose DS genes (*Clostridium* and *Francisella*).

**Fig. 5:**
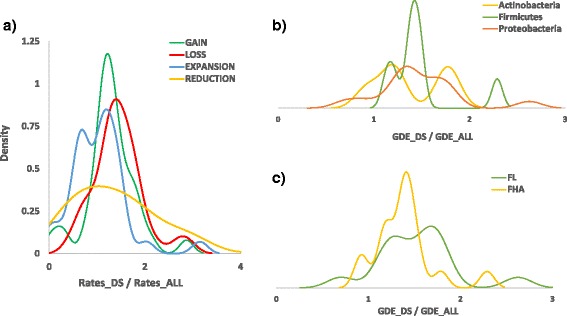
Density plots of rates of genome dynamics in defense systems normalized by the rates in all genes. **a** Distribution in all ATGCs. **b** Distribution in Actinobacteria, Firmicutes and Proteobacteria. **c** Distribution in free living (FL) and facultative host associated (FHA) bacteria

On average, the rates of DS gene dynamics per COG are about 1.4 times higher than the rates for all genes (the ratios are, approximately: 1.5 for gene loss, 1.3 for gene gain, 1.5 for gene family reduction, and 1.04 for gene family expansion) (Fig. 5a and Table 1). The difference between the rates for DS and the means over all functional categories of genes are statistically significant for the total of all GDE, loss and gain (*p*-values of 0.0056, 0.0071 and 0.0284, respectively) as calculated from the differences in the log-likelihoods (see Methods for details). These ratios vary within a broad range, from nearly absent gene gain in *Chlamydia*-*Chlamydophila* to a near threefold excess of the loss rate over the genomic average in *Helicobacter*. Nevertheless, the trend of higher than average genome dynamics rates for DS is consistent: it holds for 30 of the 36 ATGCs for gene gain and 31 of the 36 ATGCs for loss (Table 1). The observed differences cannot be explained by taxonomy (Fig. 5b) or life style (Fig. 5c).

**Fig. 6:**
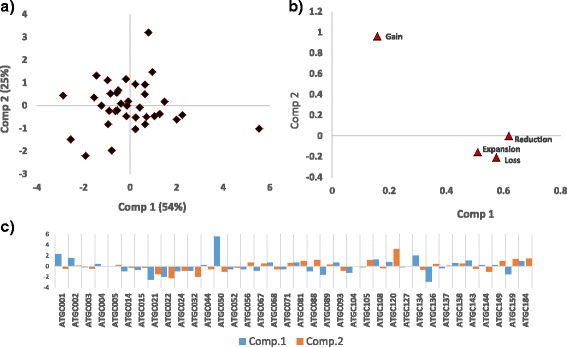
Principal components analysis of the relative rates of gain, loss, expansion and reduction in defense systems normalized by the rates in all genes. **a** PCA plot. Each dot corresponds to an ATGC. **b** Loadings plot. **c** Bar plot of the PCA’s main principal components by ATGC

In the principal component analysis (PCA), the first principal component (PC1) explains 54% of the variance and separates ATGCs with high and low gene flux in DS genes. The second principal component (PC2) explains 25% of the variance and separates the gainers of DS genes from the rest of the ATGCs (Fig. 6a, b). Predictably, the evolution of most of the ATGCs is dominated by PC1 but several ATGCs that are active gainers are primarily characterized by PC2 (Fig. 6c). *Helicobacter* shows the highest flux among the ATGCs whereas *Shewanella* presents the highest gene gain rate.

### Case by case analysis of the evolution of defense systems

We examined in detail several specific cases of gain or loss of defense-related genes in different ATGCs, in an attempt to illustrate various evolutionary trends. The ATGCs for this case by case, namely ATGC068: *Corynebacterium* (Additional file 1: Figure S1), ATGC081: *Clostridium* (Additional file 1: Figure S2) and ATGC159:*Propionibacterium* (Additional file 1: Figure S3), were selected based by the following criteria: 1) approximately 10 genomes in an ATGC; 2) high genome synteny allowing for whole genome alignments and 3) moderate number of GDE allowing for comprehensive analysis.

#### ATGC068: Corynebacterium

The ATGC068 consists of 13 closely related *Corynebacterium diphtheriae* strains. The COUNT reconstruction indicates a strong DS gene loss trend in all branches of the tree (Additional file 1: Figure S4). Below we describe several examples of loss and gain of defense genes in this ATGC. The COUNT reconstruction indicates several losses of *cas* genes during the evolution of *Corynebacterium diphtheriae* (Additional file 1: Table S6). All of these genes apparently have been lost from the genomic region between the genes for hydroxymethylpyrimidine/phosphomethylpyrimidine kinase *thiD* and serine/threonine kinase *pknB* genes (Additional file 1: Figure S5). A CRISPR-Cas system is present in this locus in each genome but interestingly, these belong to two different subtypes [34], namely II-C (in 8 genomes) and I-E (in the remaining 5 genomes). The organisms that possess the I-E system are not monophyletic in the respective species tree (Additional file 1: Figure S5). In the phylogenetic tree of Cas1 [34], the II-C and I-E Cas1 proteins from this locus form two clades within the II-C and I-E branches, respectively. Thus, the most parsimonious scenario is that type II-C is ancestral in this locus but had been replaced with I-E systems from sources within the same bacterial lineage on several independent occasions. The reversal of such a replacement, i.e. restoration of type II-C system in this locus might have occurred as well as suggested by the mapping of the cas9 gene tree onto the species tree (Additional file 1: Figure S5) which shows that Cas9 from *C. diphtheriae* VA01 is more closely related to that of *C. diphtheriae* 31A than to the Cas9 proteins from related strains on the species tree (Additional file 1: Figure S5). Transposases that are present in these loci might have facilitated recombination. This example shows that *in situ* replacements of CRISPR-Cas systems by different types might be common among related microbial strains.

**Fig. 7:**
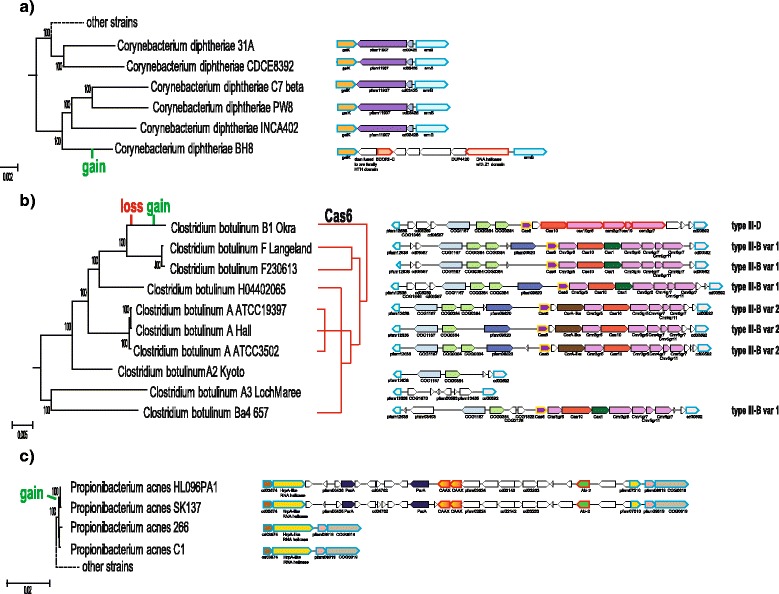
Examples of defense gene gain. The trees for the ATGC genomes, reconstructed from concatenated alignments of nucleotide sequences of common orthologs [29] are shown in the left part of each panel. Defense system loci are schematically depicted in the right part of each panel. Homologous genes are highlighted in matching colors. Genes that are rare or unique in these regions are shown as blank shapes. Genes are labeled by the gene names or by NCBI CDD profile names. Gained genes are shown by red outline. Conserved flanking genes are shown by blue outline. **a** Acquisition of RM-related genes in *Corynebacterium diphtheriae* BH8. **b** Recombination *in situ* in the CRISPR-*cas* locus of *Clostridium botulinum* strains. The *cas* genes and CRISPR-Cas system subtypes are labeled according to the current CRISPR-Cas system classification and nomenclature [34]. The *cas6* gene not affected by recombination are is highlighted by yellow outline. The phylogenetic tree for the *cas6* nucleotide sequences is schematically shown opposite the genome tree. **c** Acquisition of Abi genes in the *Propionibacterium acnes* strains SK137 and HL096PA1

Two RM genes, a EcoRII-like restriction endonuclease and a diverged predicted helicase containing a Z1 domain previously found to be associated with RM systems [35, 36], were inferred to have been gained on the terminal branch corresponding to *C. diphtheriae* BH8 (Fig. 7a). These two genes belong to an island of 6 singletons, i.e. genes restricted to a single genome within the analyzed data set, (CDBH8_0988-CDBH8_0994) inserted between galactokinase *galK* and RNA helicase *srmB* genes. In addition to the two RM genes, the island encodes a Dcm-like cytosine-C5 specific DNA methylase fused to a Xre family HTH (helix-turn-helix) domain, a DUF4420 (pfam14390) and three unclassified proteins (Fig. 7a). The best BLASTP matches for these proteins in the NR database are to homologs from other *Corynebacterium* species (although none from this ATGC) but the similarity is comparatively low, around 40% identity (data not shown), suggesting acquisition of this region via HGT from a relatively distant bacterium. This island replaces two genes that are present in all other genomes from this ATGC, namely a Superfamily II DNA helicase fused to a phospholipase D family nuclease domain (pfam11907) and a MutT-like pyrophosphohydrolase (cd03425). The latter pair of genes is missing in the genome of *C. diphtheriae* BH8 suggesting that it was lost in the process of the island integration. Despite the presence of two genes that have been previously identified in the context of known RM systems, no full gene complement for any characterized type of RM systems could be identified in this region. Thus, either these proteins are functionally unrelated and are encoded together in a defense island by chance or some subset of the genes in the island comprises a novel, multicomponent defense system.

The COUNT reconstruction predicts a gain of an Abi2 (HEPN domain-containing protein, predicted ribonuclease) gene in *C. diphtheriae* VA01. This gene is absent from all other genomes of this ATGC and shows the highest similarity to homologs from *Corynebacterium pseudotuberculosis* suggesting acquisition of this gene from a more distant species of the same genus. The region upstream of the superoxide dismutase gene, where the Abi2 gene apparently was inserted, shows variable gene content. In *C. diphtheriae* VA01, it contains several uncharacterized genes some of which encode predicted DS proteins such as three subunits of a Type I RM system, a PD-(D/E)xK nuclease and Fic/DOC, and HTH domain-containing protein shared with several other species from this ATGC (Additional file 1: Figure S6). Three genes in this region (CDVA01_2131- CDVA01_2133) including one for a PD-(D/E)xK nuclease (CDVA01_2132, often annotated as “P51 protein”) are found next to each other in Gram-positive bacterial prophages of the phi-APSE family although no other phage-associated genes were identified in this region. Nevertheless, the presence in this neighborhood of a gene encoding a ParA family ATPase known to be involved in plasmid partitioning [37] implies that this region is associated with a mobile element.

In a case of apparent loss of a TA gene pair (a toxin containing a truncated PIN domain, apparently an inactived ribonuclease, and an antitoxin containing a Xre family HTH domain) in *Corynebacterium diphtheria* VA01, the two genes apparently were excised together with a mobile element (as inferred by the presence of an integrase gene in this region), which had integrated next to the TA module in the ancestor of the three related *C. diphtheriae* strains (Additional file 1: Figure S7). Thus, this case is an example of mobilization of chromosomal genes. In the corresponding region in several other genomes that lack the mobile element, the toxin had been independently truncated, whereas the antitoxin remained intact, suggesting that toxin inactivation could be a general trend in evolution of toxin-antitoxin modules (Additional file 1: Figure S7).

#### ATGC081: *Clostridium botulinum*

The ATGC081 contains 10 closely related *Clostridium botulinum* strains. This ATGC also shows an overall tendency to lose DS genes according to the COUNT prediction, from 49 at the root to 30–35 at the tips (Additional file 1: Figure S8).

Four DS genes predicted to have been gained in *Clostridium botulinum* A2 str Kyoto belong to a genomic island (CLM_2285- CLM_2304) between Radical SAM superfamily protein and UDP-N-acetylglucosamine 4-epimerase genes (Additional file 1: Figure S9). These four DS genes encode an AIPR family Abi gene and three subunits of a type I restriction modification system (Additional file 1: Figure S9). In addition, the island encodes two proteins that are common in phages, namely a holin (CLM_2290) and an N-acetylmuramoyl-L-alanine amidase (CLM_2289), suggesting that this region is of a phage origin. This region shows considerable variability in other genomes from this ATGC (Additional file 1: Figure S9).

A replacement of a type III-B CRISPR-*cas* locus with type III-D is observed in *Clostridium botulinum* B1 Okra (Fig. 7b). The replacement apparently occurred *in situ,* without affecting the c*as6* gene, which is present in this region in all Clostridium botulinum species from this ATGC. These *cas6* sequences are highly similar on the nucleotide level, whereas even on the amino acid sequence level, there is only weak similarity between the Cas6 proteins of subtypes III-D and III-B. The Cas6 protein is an endoribonuclease that is required for pre-crRNA transcript processing and is often encoded in the type III loci, even those that lack a CRISPR array [34]. This particular III-D locus is not found in any of the *C. botulinum* species and shows the closest similarity to the III-D locus of *Clostridium lundense*, suggesting horizontal transfer from a relatively distant species. Furthermore, the phylogeny of the Cas10 protein [34] suggests that at least one additional *in situ* gene replacement occurred in this clade because Cas10 and, as we show here, the respective gene set of type III-B systems are distinct in the strains of *C. botulinum* strains ATCC 19397, ATCC 3502 and Hall, and in the branch that encompasses strains H04402 065, F str. 230,613 and F str. Langeland. In contrast, *cas6* genes can be confidently aligned at the nucleotide level and thus apparently were unaffected by these events (Fig. 7b)*.*

#### ATGC159: Propionibacterium

The ATGC159 consists of 10 closely related *Propionibacterium acnes* strains and one strain of *Propionibacterium avidum*. Based on the COUNT estimates, the *P. acnes* strains have lost DS genes (from 20 at the root to 11–18 at the tips) whereas *P. avidum* gained 9 additional DS genes (Additional file 1: Figs. S3 and S10).

The gain of several Abi genes (one Abi2 family genes and two CAAX family protease genes) most likely occurred due to the insertion of a large DNA segment containing 23 genes (PAGK_0160- PAGK_0182 locus in *P. acnes* HL096PA1 (for details, see Additional file 1: Figure S11) into the genome of the common ancestor of *P. acnes* SK137 and *P. acnes* HL096PA1 (Fig. 7c). This DNA fragment was inserted into the HrpA-like RNA helicase gene which was disrupted in the process. There are two ParA ATPase genes encoded in this locus (Fig. 7c). The DNA-binding ATPase ParA, typically together with another DNA-binding protein, ParB, is essential for plasmid partitioning [38]. Although the *parB* gene is missing in *P. acnes,* the presence of the *parA* genes suggests that this genomic segment derives from a plasmid.

Another example of DS gene gain also involves acquisition of a large genomic island (PAC1_03830-PAC1_03980) in *P. acnes* C1 (Additional file 1: Figure S12). The island includes a toxin gene of the Fic/Doc family (PAC1_03955) that is located next to a gene encoding an antitoxin of the VbhA family [39]. Recently, an antitoxin of this family has been shown to repress growth arrest mediated by the FIC domain-containing toxin VbhT in the mammalian pathogen *Bartonella schoenbuchensis* [39]. In *P. acnes*, the island is inserted into an alpha-beta family hydrolase gene and is flanked by transposases. It includes a number of genes unrelated to defense but implicated in stress response, particularly metal resistance (Additional file 1: Figure S12). We were unable to identify an identical or substantially similar island in any other complete genome present in our data set but the presence of a ParA family ATPase and the transposases again suggests that the island could be a mobile element.

#### Distribution of defense systems and genome dynamic events on bacterial chromosomes

Given the previous observations on defense islands in microbial genomes [36], we examined possible clustering of defense genes in the ATGC genomes. Defense genes show a significant trend of co-localization in 57% of the genomes (Additional file 1: Figure S13a), for all classes of defense systems (Additional file 1: Figure S13b). However, when the analysis was repeated with “directons”, i.e. groups of co-directed genes that comprise putative operons [40], random distribution of DS was observed in 83% of the genomes (Additional file 1: Figure S13a (Additional file 1: Figure S13c). Additionally, we analyzed in detail the distribution of inferred DS gene gains and losses in the chromosomes of ATGC068 (*Corynebacterium*), ATGC081 (*Clostridium*) and ATGC159 (*Propionibacterium*). In 10 of the 18 analyzed genomes (14 genomes for directons), gains and losses were found to be randomly distributed across the chromosome (Additional file 1: Figure S14).

## Conclusions

The results of the present reconstruction of the evolution of defense systems in prokaryotes perhaps can be considered “disappointing” in that no distinct evolutionary regime was discovered for the defense genes. The evolution of the DS is consistently and significantly more dynamic than the genomic mean for the respective groups of microbes. However, the difference in the GDE rates is moderate (less than 1.5 fold), perhaps surprisingly, given the common view of the evolution of defense systems in the regime of incessant arms race with parasites [41–43]. The evolution of defense systems is shaped by several factors that seem to exert different, in some cases opposite effects on the gain and loss rates of defense genes. The arms race is only one of such factors. The others include the fitness cost of defense systems stemming from energetic burden, autoimmunity and barriers to horizontal gene transfer and potentially enhancing loss over gain [44, 45]; the selfish behavior of defense system, particularly RM and TA, which often become addictive to the host cells, such that loss is inhibited, and are frequently transferred on plasmids, enhancing gain [46, 47]; and additional, non-defense functions of some of these systems, e.g. CRISPR-Cas [48], which favor retention of the respective genes over loss. It appears that the net outcome of these distinct effects constrains the mobility of defense systems, resulting in the moderate excess of the GDE rates over the genomic averages.

The relative contributions of gene family loss, gain, expansion and contraction are, on average, the same as they are for the rest of microbial genes. Furthermore, with the exception of some parasitic bacteria that encode few DS and show low flux of defense genes, there is no obvious connection between the dynamics of the DS and the life style of the respective organisms. Thus, apparently, the evolutionary dynamics of the DS follows the general trends of microbial genome evolution. These trends seem to stem from the inherent deletion bias of genome evolution combined with the neutral, largely clock-like mode of gene loss which contrasts the less uniform and partially adaptive mode of gene gain [7, 8, 49, 50]. Although the quantitative trends in the evolution of the DS seem to recapitulate the overall tendencies of microbial genome evolution, case by case analysis points to notable phenomena, such as frequent involvement of mobile elements and *in situ* replacement of the DS, particularly for different types of CRISPR-Cas systems. These features appear to reflect the frequent localization of the DS in defense islands that are enriched also in mobile elements and are likely to be responsible for the enhanced dynamics of DS evolution.

## Methods

### ATGC dataset

Genomic data were obtained from an updated version of the ATGC database [30] that contains alignable (>85% conserved synteny) and tight (synonymous substitution rate < 1.5) genomic clusters. We selected only ATGC clusters that contain at least 10 genomes. Complete genome alignments of three ATGCs (ATGC068, ATGC081 and ATGC159) were performed with the program MAUVE [51].

### Defense systems

A list of COGs, Pfam and CDD domains involved in defense systems was constructed from the results of previous studies [11, 32], including restriction-modification (RM), toxin-antitoxin (TA), and abortive infection (AI) systems, and the CRISPR-Cas systems of adaptive immunity. All defense genes were mapped onto the ATGCs using a custom Perl script and the overlapping data set was used to quantify rates of gene dynamics. In order to harness sufficient analytical power, only defense systems with more than 10 genes and more than 10 events (including gain, loss, expansion or reduction) were analyzed.

### Species trees

The program COUNT [33], which was employed for evolutionary reconstruction (see description bellow), requires rooted phylogenetic trees as an input. Thus, a species tree of each ATGC was reconstructed from a concatenated alignment of all universal genes with conserved synteny among species. Protein sequences were aligned with MUSCLE [52] and back translated to respective nucleotide sequences using an in-house script. All alignments of genes were concatenated in a single alignment and used to reconstruct a species tree for each ATGC using the program FastTree [53] under the General Time Reversible (GTR), Among Site Rate Variation (gamma) nucleotide substitution model [54]. Accordingly, all species trees were rooted using the least-squares modification of the mid-point method [55].

### Phylogenetic birth-and-death analysis

We estimated the rates of gain, loss, expansion and reduction based on a phylogenetic birth-and-death model implemented in the program COUNT [33]. The program estimates the rates of gene gain (κ), individual gene duplication (λ) and individual gene loss (μ). Thus, a gene family of size n decreases at a rate *nμ* and increases at a rate (κ + nλ). The parameters (κ,λ,μ) are different for each gene family and across edges of the species tree. The parameters were optimized iteratively, as recommended [33], as previously described [7]. Thus, the parameters were optimized in each ATGC individually (using all gene families) through 10 rounds of increasing complexity, from uniform rates of gain, loss and duplication to up to 4 discrete categories for the gamma distribution. The parameter values obtained in the final round were used to estimate the numbers of gains, losses, expansions and reductions at different branches of the species tree for gene families involved in defense systems. The final number of events for each ATGC was estimated as the sum over all branches and across all families. The *p*-values for the differences in the rates of gene dynamics events between DS and the rest of the genes were calculated from the differences in log-likelihoods using the Welch Two Sample t-test implemented in R.

### Principal component analysis

The input variables for the PCA were the relative rates of gain, loss, expansion and reduction in defense systems in each ATGC (Table 1). These values were transformed into the logarithmic scale prior to the analysis. The PCA was performed using the function *princomp* from the R statistical package.

### Distribution of genes and dynamic events in the chromosome

We analyzed all genomes from each ATGC individually to assess whether DS genes are randomly distributed in the chromosome (Additional file 1: Figure S15). First, we calculated the median distance of each DS gene to the closest DS gene. Then, we performed a randomization test, randomly sampling the same number of genes on each chromosome as there are defense genes and calculating the median distance between sampled genes. Repeating this procedure 10,000 times allowed us to test the null hypothesis that DS genes are not closer together than chance expectations. Alternatively, directons (i.e. strings of consecutive co-directed genes [40]) were marked on each chromosome and labeled as defense directons if at least one of the genes in a directon belongs to DS. Then the median distance between the defense directons was compared to that between randomly sampled directons as described above.

Similarly, the distribution of gains and losses of DS genes on the terminal branches in ATGC068, ATGC081 and ATGC159 was analyzed by mapping these events on the corresponding chromosomes and calculated the median distance between these locations. For comparison, the same number of genes were randomly sampled from all defense genes on these chromosomes and the median distance calculated; the procedure was repeated 10,000 times. The test was performed with both individual genes and at the directon level.

#### Software availability

All custom scripts used for this analysis are available at ftp://ftp.ncbi.nlm.nih.gov/pub/wolf/_suppl/ATGCdefense.

## Additional file

Additional file 1: Figure S1. Gains and losses in ATGC068-*Corynebacterium*. **Figure S2.** Gains and losses in ATGC081-*Clostridium*. **Figure S3.** Gains and losses in ATGC159-*Propionibacterium*. **Figure S4.** Count output for ATGC068-*Corynebacterium*. **Figure S5.** Example of multiple independent substitutions of CRISPR-Cas system type II-C to type I-E. **Figure S6.** Example of predicted gain by COUNT of Abi2 gene in a large loci with multiple gains and losses. **Figure S7.** Example of TA gene loss. **Figure S8.** Count output for ATGC081-*Clostridium*. **Figure S9.** Example of gain of four DS genes in *Clostridium botulinum* A2 str Kyoto. **Figure S10.** Count output for ATGC159-*Propionibacterium*. **Figure S11.** Locus details for Fig. 7c. **Figure S12.** Example of TA gene gain (within a large locus) in *Propionibacterium acnes* C1. **Figure S13.** Density distribution of *p*-values from the randomization test. **Figure S14.** Distribution of defense systems and dynamic events in 18 genomes. **Figure S15**. Scheme of the methodology used to test randomness in the distribution defense genes and dynamic events in the chromosome. **Table S1.** Distribution of defense systems COGs in ATGCs. **Table S2.** Number of the defense systems normalized by the total number of genes (COGs). **Table S3.** Genome dynamics in defense systems, including gain, loss, expansion and reduction: (a) total number of events; (b) events relative to the number of COGs and (c) events relative to the number of COGs and genomes. Defense systems with less than 10 genes or less than 10 events are left empty. **Table S4.** Comparison of the genome dynamics in defense systems (relative to the dynamics in all genes) between life styles using the Welch Two Sample t-test implemented in R. **Table S5.** Comparison of the genome dynamics in defense systems (relative to the dynamics in all genes) between taxa using the Welch Two Sample t-test implemented in R. **Table S6.** Relative fluxes in defense systems. **Table S7.** Description of genes in Additional file 1: Figures. S1, S2 and S3. **Table S8.** Locus description of Additional file 1: Figure S9. **Table S9.** Locus description of Additional file 1: Figure S12. **Table S10.** *P*-values of Additional file 1: Figure S14. (DOCX 2297 kb)

## Abbreviations

AI: Abortive infection
ATGC: Alignable Tight Genome Clusters
CRISPR-Cas: Clustered Regularly Interspaced Repeats and CRISPR-associated (proteins)
DS: Defense systems
HGT: Horizontal gene transfer
RM: Restriction-modification
TA: Toxin-antitoxin
