# Supplementary material for: Reconstruction of the evolution of microbial defense systems
Source: BMC Evol Biol. 2017 Apr 4;17:94. doi: 10.1186/s12862-017-0942-y (PMC5379612; doi:10.1186/s12862-017-0942-y)
Supplement: Additional file 1: Figure S1. — Gains and losses in ATGC068-Corynebacterium. Figure S2. Gains and losses in ATGC081-Clostridium. Figure S3. Gains and losses in ATGC159-Propionibacterium. Figure S4. Count output for ATGC068-Corynebacterium. Figure S5. Example of multiple independent substitutions of CRISPR-Cas system type II-C to type I-E. Figure S6. Example of predicted gain by COUNT of Abi2 gene in a large loci with multiple gains and losses. Figure S7. Example of TA gene loss. Figure S8. Count output for ATGC081-Clostridium. Figure S9. Example of gain of four DS genes in Clostridium botulinum A2 str Kyoto. Figure S10. Count output for ATGC159-Propionibacterium. Figure S11. Locus details for Fig. 7c. Figure S12. Example of TA gene gain (within a large locus) in Propionibacterium acnes C1. Figure S13. Density distribution of p-values from the randomization test. Figure S14. Distribution of defense systems and dynamic events in 18 genomes. Figure S15. Scheme of the methodology used to test randomness in the distribution defense genes and dynamic events in the chromosome. Table S1. Distribution of defense systems COGs in ATGCs. Table S2. Number of the defense systems normalized by the total number of genes (COGs). Table S3. Genome dynamics in defense systems, including gain, loss, expansion and reduction: (a) total number of events; (b) events relative to the number of COGs and (c) events relative to the number of COGs and genomes. Defense systems with less than 10 genes or less than 10 events are left empty. Table S4. Comparison of the genome dynamics in defense systems (relative to the dynamics in all genes) between life styles using the Welch Two Sample t-test implemented in R. Table S5. Comparison of the genome dynamics in defense systems (relative to the dynamics in all genes) between taxa using the Welch Two Sample t-test implemented in R. Table S6. Relative fluxes in defense systems. Table S7. Description of genes in Additional file 1: Figures. S1, S2 and S3. Table S8. Locus description of Ad [file 12862_2017_942_MOESM1_ESM.docx]

**Additional file 1**

**
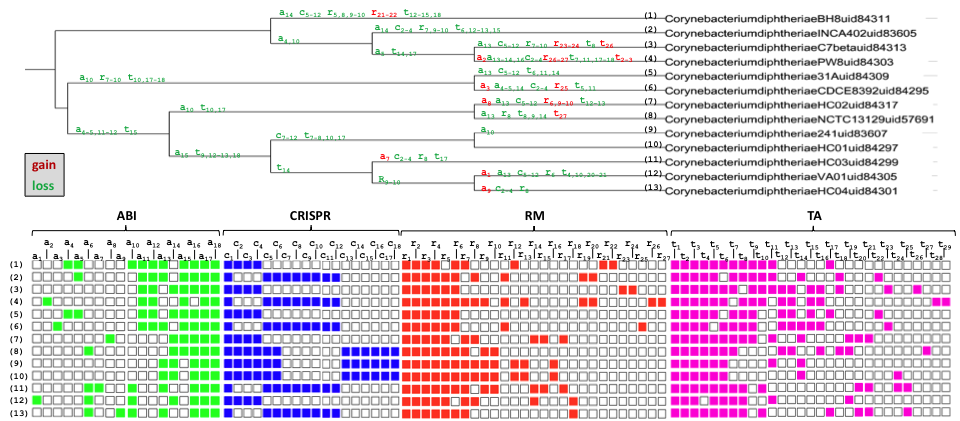
**

**Figure S1. Gains and losses in ATGC068-*Corynebacterium***

Gene descriptions can be found in supplementary table 7


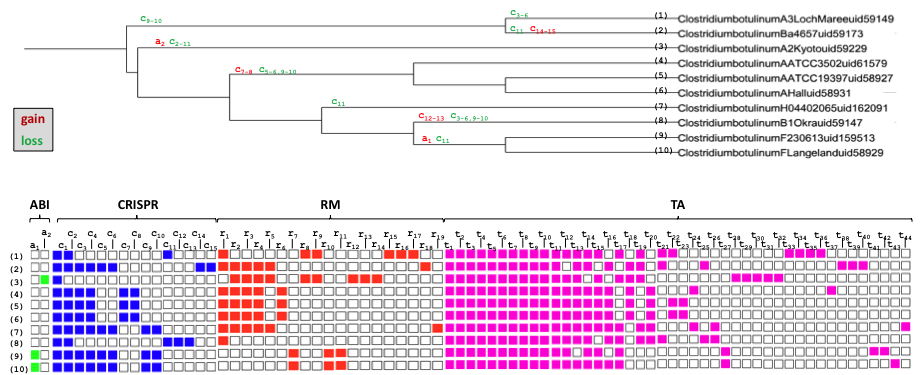


**Figure S2. Gains and losses in ATGC081-*Clostridium***

Gene descriptions can be found in supplementary table 7


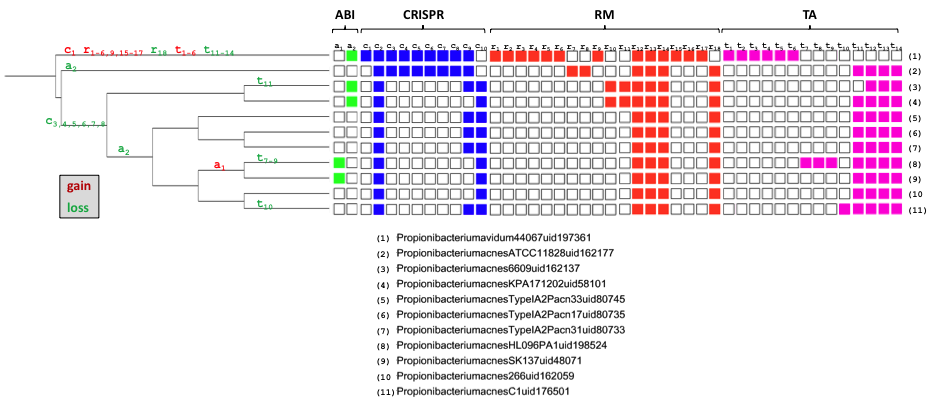


**Figure S3. Gains and losses in ATGC159-*Propionibacterium***

Gene descriptions can be found in supplementary table 7


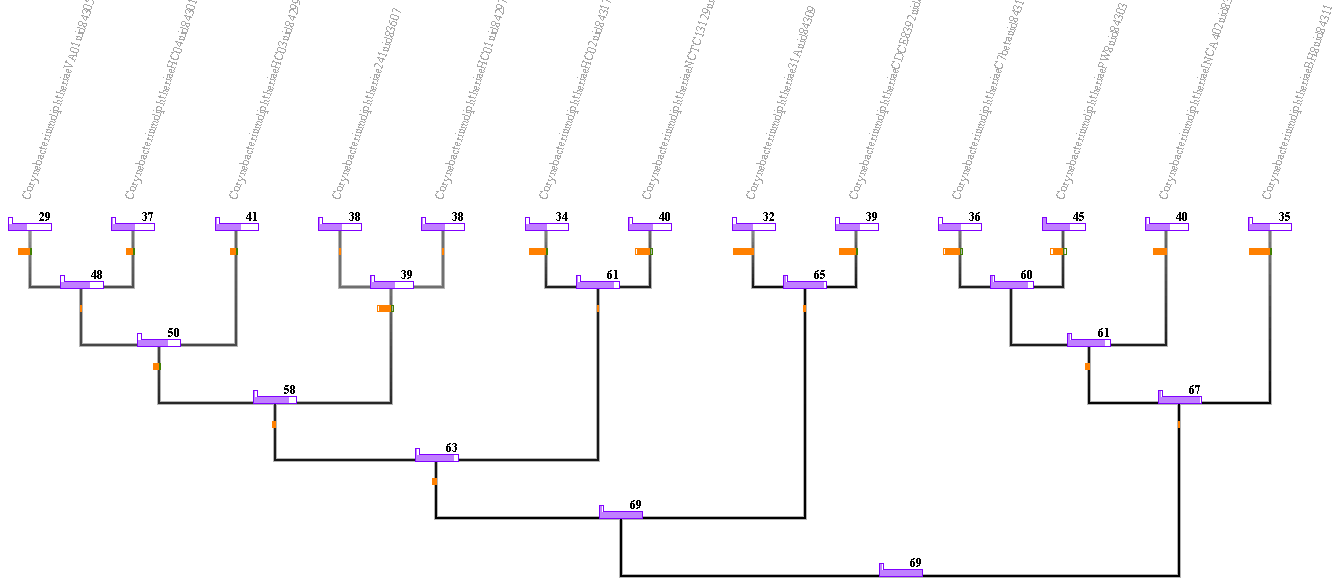


**Figure S4. Count output for ATGC068-*Corynebacterium***

Numbers and purple bars correspond to the real (at the tips) and estimated (in the branches) number of DS genes. Green and orange bars correspond to gain and loss of genes.


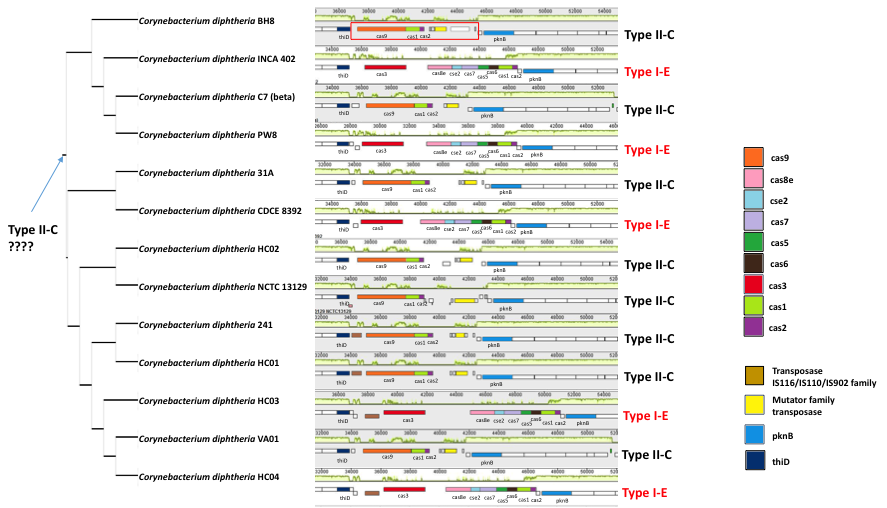


**Figure S5. Example of multiple independent substitutions of CRISPR-Cas system type II-C to type I-E**

The species tree for the ATGC068-*Corynebacterium*, reconstructed from concatenated alignments of nucleotide sequences of common orthologs [[1](#_ENREF_1)], is shown on the left. Defense system loci are shown on the right. Homologous genes are highlighted in matching colors.


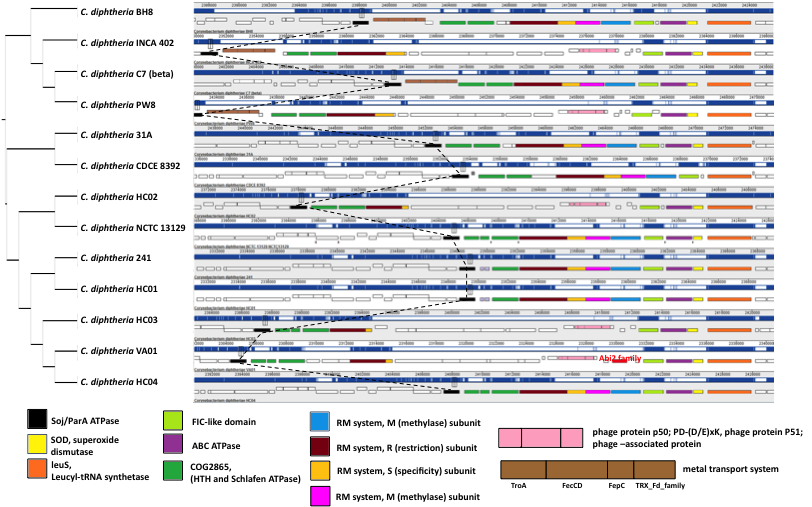


**Figure S6. Example of predicted gain by COUNT of Abi2 gene in a large loci with multiple gains and losses**

The species trees for the ATGC068-*Corynebacterium*, reconstructed from concatenated alignments of nucleotide sequences of common orthologs [[1](#_ENREF_1)], is shown on the left. Defense system loci are shown on the right. Homologous genes are highlighted in matching colors.


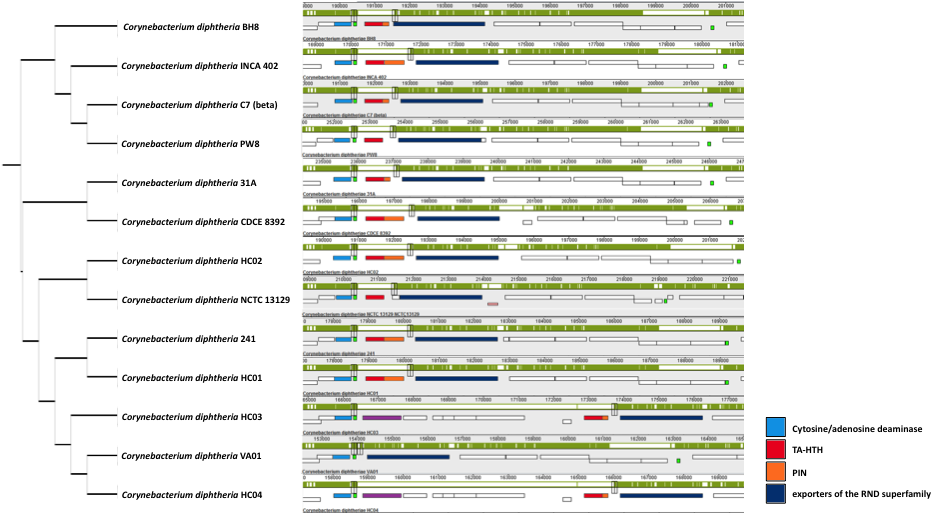


**Figure S7. Example of TA gene loss**

The species trees for the ATGC068-*Corynebacterium*, reconstructed from concatenated alignments of nucleotide sequences of common orthologs [[1](#_ENREF_1)], is shown on the left. Defense system loci are shown on the right. Homologous genes are highlighted in matching colors.

**
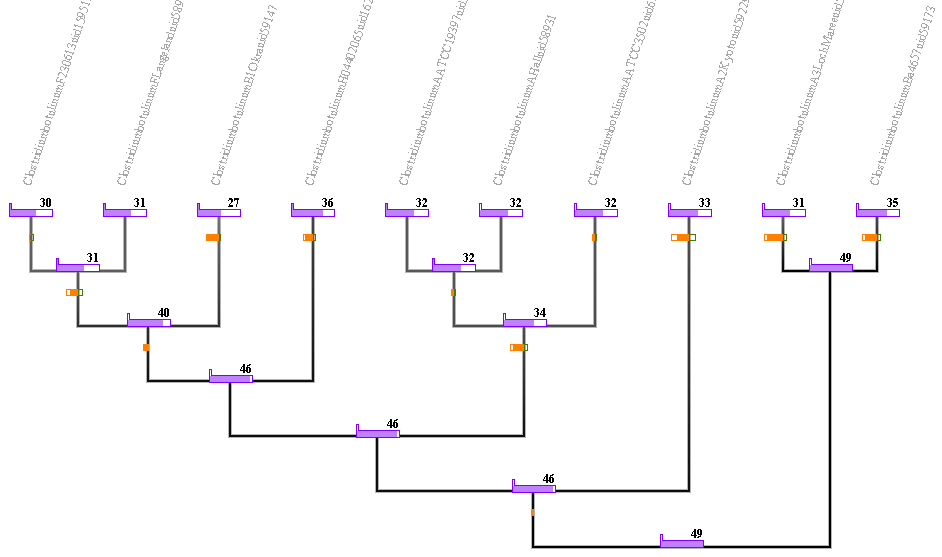
**

**Figure S8. Count output for ATGC081-*Clostridium***

Numbers and purple bars correspond to the real (at the tips) and estimated (in the branches) number of DS genes. Green and orange bars correspond to gain and loss of genes.


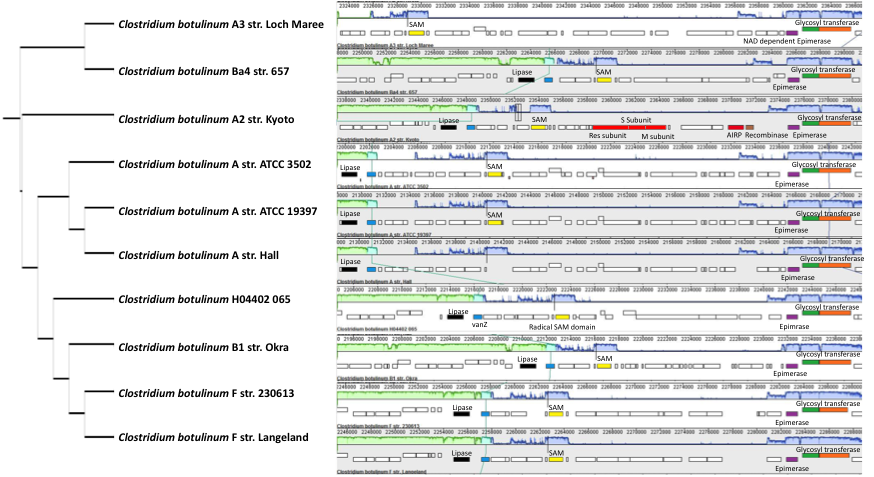


**Figure S9. Example of gain of four DS genes in *Clostridium botulinum* A2 str Kyoto**

The species trees for the ATGC081-*Clostridium*, reconstructed from concatenated alignments of nucleotide sequences of common orthologs [[1](#_ENREF_1)], is shown on the left. Defense system loci are shown on the right. Homologous genes are highlighted in matching colors. Locus description for *Clostridium botulinum* A2 str. Kyoto in supplementary table 8


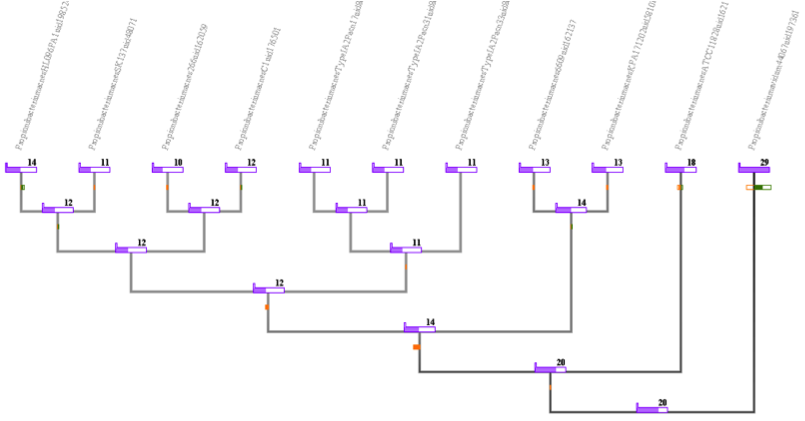


**Figure S10. Count output for ATGC159-*Propionibacterium***

Numbers and purple bars correspond to the real (at the tips) and estimated (in the branches) number of DS genes. Green and orange bars correspond to gain and loss of genes.


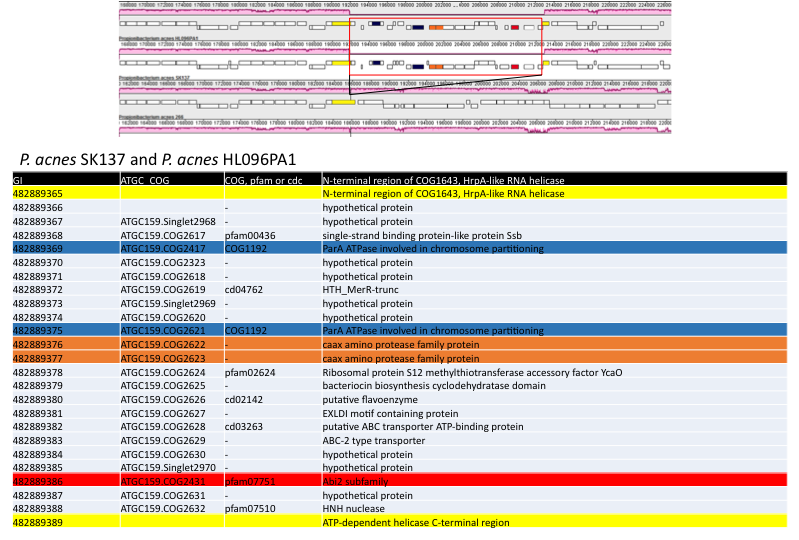


**Figure S11. Locus details for Figure 7c**


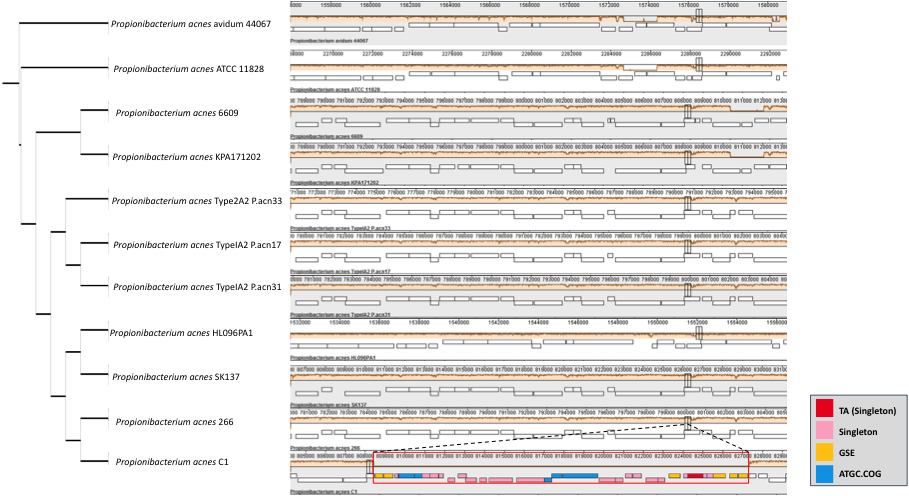


**Figure S12. Example of TA gene gain (within a large locus) in *Propionibacterium acnes* C1**

The species trees for the ATGC159-Propionibacterium, reconstructed from concatenated alignments of nucleotide sequences of common orthologs [[1](#_ENREF_1)], is shown on the left. Defense system loci are shown on the right. TA gene highlighted in red, the rest of genes of *P. acne* C1 are highlighted according ATGC descriptors (COGs in blue, GSE in yellow and singletons in pink). Full Locus description for *P. acne* C1 in supplementary table S9.

Homologous genes are highlighted in matching colors.


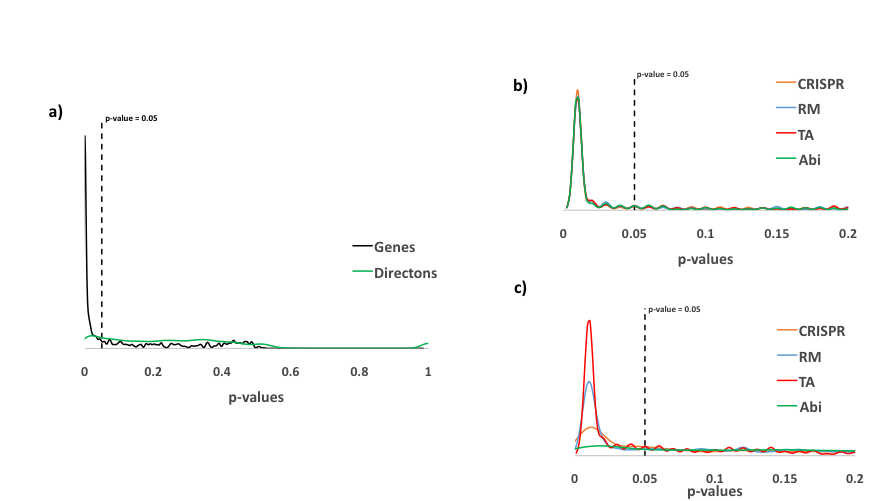


**Figure S13. Density distribution of p-values from the randomization test**

a) P-values from the randomization test of all genes (black line) and directons (green line)

b) P-values from the randomization test of genes (CRISPR, orange line; RM, blue line; TA, red line; Abi, green line)

c) P-values from the randomization test of directons (CRISPR, orange line; RM, blue line; TA, red line; Abi, green line)


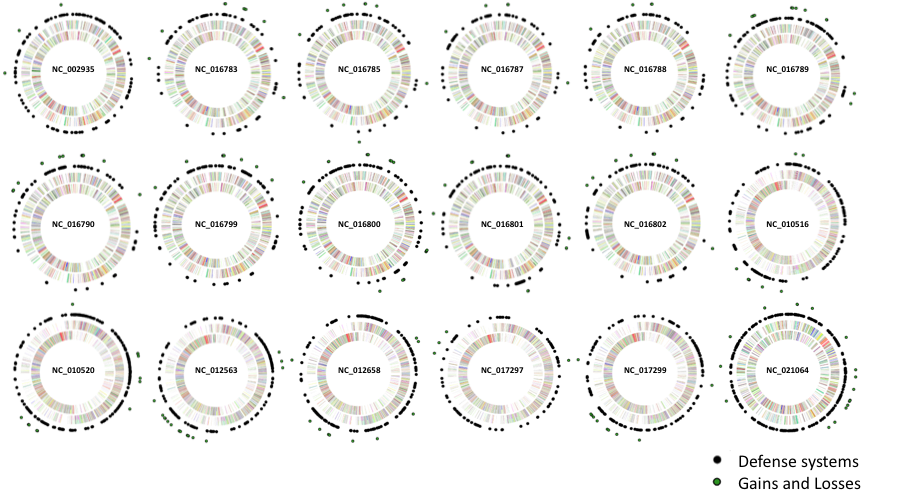


**Figure S14. Distribution of defense systems and dynamic events in 18 genomes.**

P-values are included in supplementary table S10.


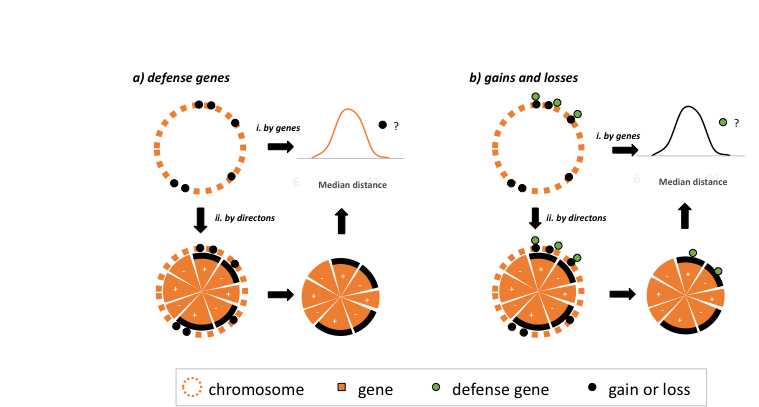


**Figure S15. Scheme of the methodology used to test randomness in the distribution defense genes and dynamic events in the chromosome**

1. Defense genes. (i) Randomness in defense genes is assessed through comparison of the median distance between closest defense genes and a random distribution. This distribution is created by randomly sampling as many genes as defense genes and then calculate the median distance between genes (10,000 replications). (ii) Same procedure, but genes are previously grouped together in directons (see Methods).

b) Gains and losses. In this case, the median distance is calculated between closest gains or losses and compared to a random distribution calculated from defense genes; same procedure as in (a).

**Table S1. Distribution of defense systems COGs in ATGCs**

| **ATGC** | **Genera** | **N_SP** | **N_COGs** | **Abi** | **CRISPR** | **RM** | **RM_DND** | **TA** | **Unknown** |
| --- | --- | --- | --- | --- | --- | --- | --- | --- | --- |
| **ATGC149** | **Acinetobacter** | 14 | 6457 | 4 | 14 | 21 | 1 | 26 | 35 |
| **ATGC014** | **Bacillus** | 31 | 16679 | 16 | 33 | 43 | 4 | 93 | 44 |
| **ATGC015** | **Bacillus** | 24 | 9243 | 4 | 6 | 26 | 1 | 29 | 20 |
| **ATGC104** | **Bifidobacterium** | 11 | 4353 | 11 | 23 | 26 | 0 | 53 | 40 |
| **ATGC105** | **Bifidobacterium** | 10 | 2019 | 0 | 18 | 5 | 1 | 19 | 14 |
| **ATGC144** | **Borrelia** | 11 | 1922 | 0 | 0 | 4 | 0 | 1 | 42 |
| **ATGC136** | **Brucella-Ochrobactrum** | 19 | 6014 | 3 | 4 | 11 | 0 | 35 | 44 |
| **ATGC088** | **Burkholderia** | 13 | 13383 | 5 | 13 | 34 | 0 | 45 | 53 |
| **ATGC089** | **Burkholderia** | 12 | 14542 | 12 | 19 | 32 | 4 | 119 | 97 |
| **ATGC143** | **Campylobacter** | 11 | 2638 | 2 | 5 | 17 | 0 | 23 | 15 |
| **ATGC044** | **Candidatus-Rickettsia** | 40 | 4456 | 2 | 5 | 12 | 0 | 91 | 20 |
| **ATGC021** | **Chlamydia** | 45 | 1218 | 0 | 2 | 2 | 0 | 2 | 7 |
| **ATGC022** | **Chlamydia-Chlamydophila** | 21 | 1536 | 0 | 2 | 0 | 0 | 2 | 8 |
| **ATGC001** | **Enterobacteria** | 109 | 24846 | 25 | 40 | 89 | 6 | 159 | 142 |
| **ATGC081** | **Clostridium** | 10 | 5992 | 4 | 32 | 19 | 0 | 44 | 20 |
| **ATGC067** | **Corynebacterium** | 18 | 3255 | 2 | 25 | 13 | 0 | 16 | 9 |
| **ATGC068** | **Corynebacterium** | 13 | 3560 | 19 | 25 | 28 | 0 | 31 | 21 |
| **ATGC002** | **Enterobacter-Klebsiella** | 11 | 10803 | 12 | 28 | 46 | 2 | 71 | 69 |
| **ATGC138** | **Francisella** | 18 | 3253 | 3 | 9 | 19 | 0 | 14 | 11 |
| **ATGC050** | **Helicobacter** | 51 | 4569 | 9 | 8 | 78 | 0 | 22 | 27 |
| **ATGC056** | **Lactobacillus** | 10 | 4894 | 5 | 21 | 19 | 1 | 21 | 23 |
| **ATGC184** | **Legionella** | 10 | 4588 | 15 | 19 | 14 | 0 | 38 | 36 |
| **ATGC108** | **Listeria** | 31 | 5835 | 7 | 21 | 25 | 0 | 21 | 24 |
| **ATGC024** | **Mycobacterium** | 32 | 7294 | 14 | 34 | 19 | 0 | 124 | 33 |
| **ATGC032** | **Mycoplasma** | 11 | 884 | 0 | 4 | 3 | 0 | 0 | 4 |
| **ATGC137** | **Neisseria** | 18 | 4345 | 3 | 23 | 35 | 0 | 42 | 31 |
| **ATGC159** | **Propionibacterium** | 11 | 3486 | 2 | 12 | 21 | 0 | 14 | 17 |
| **ATGC071** | **Pseudomonas** | 12 | 11390 | 10 | 17 | 36 | 1 | 96 | 80 |
| **ATGC120** | **Shewanella** | 14 | 8091 | 13 | 29 | 50 | 3 | 80 | 75 |
| **ATGC052** | **Staphylococcus** | 42 | 4846 | 3 | 17 | 23 | 2 | 24 | 64 |
| **ATGC003** | **Streptococcus** | 22 | 4480 | 10 | 6 | 29 | 0 | 38 | 18 |
| **ATGC004** | **Streptococcus** | 22 | 3747 | 4 | 15 | 13 | 0 | 23 | 42 |
| **ATGC005** | **Streptococcus** | 16 | 3467 | 7 | 12 | 27 | 0 | 31 | 26 |
| **ATGC093** | **Sulfolobus** | 12 | 4472 | 5 | 87 | 13 | 0 | 84 | 24 |
| **ATGC134** | **Xanthomonas** | 13 | 9817 | 7 | 22 | 41 | 2 | 72 | 65 |
| **ATGC127** | **Yersinia** | 19 | 8289 | 3 | 16 | 24 | 0 | 78 | 52 |

** N_COGs = Number of COGs (including all genes) in the ATGC; N_SP = Number of species in the ATGC*

**Table S2. Number of the defense systems normalized by the total number of genes (COGs)**

| **ATGC** | **Abi** | **CRISPR** | **RM** | **RM_DND** | **TA** | **Unknown** | **p-value (chi-test)** |
| --- | --- | --- | --- | --- | --- | --- | --- |
| **ATGC001** | 0.101 | 0.161 | 0.358 | 0.024 | 0.640 | 0.572 | 0.2243 |
| **ATGC002** | 0.111 | 0.259 | 0.426 | 0.019 | 0.657 | 0.639 | 0.2243 |
| **ATGC003** | 0.223 | 0.134 | 0.647 | 0.000 | 0.848 | 0.402 | 0.2243 |
| **ATGC004** | 0.107 | 0.400 | 0.347 | 0.000 | 0.614 | 1.121 | 0.2243 |
| **ATGC005** | 0.202 | 0.346 | 0.779 | 0.000 | 0.894 | 0.750 | 0.2243 |
| **ATGC014** | 0.096 | 0.198 | 0.258 | 0.024 | 0.558 | 0.264 | 0.2243 |
| **ATGC015** | 0.043 | 0.065 | 0.281 | 0.011 | 0.314 | 0.216 | 0.2243 |
| **ATGC021** | 0.000 | 0.164 | 0.164 | 0.000 | 0.164 | 0.575 | 0.2851 |
| **ATGC022** | 0.000 | 0.130 | 0.000 | 0.000 | 0.130 | 0.521 | 0.2851 |
| **ATGC024** | 0.192 | 0.466 | 0.260 | 0.000 | 1.700 | 0.452 | 0.2243 |
| **ATGC032** | 0.000 | 0.452 | 0.339 | 0.000 | 0.000 | 0.452 | 0.2851 |
| **ATGC044** | 0.045 | 0.112 | 0.269 | 0.000 | 2.042 | 0.449 | 0.2243 |
| **ATGC050** | 0.197 | 0.175 | 1.707 | 0.000 | 0.482 | 0.591 | 0.2243 |
| **ATGC052** | 0.062 | 0.351 | 0.475 | 0.041 | 0.495 | 1.321 | 0.2243 |
| **ATGC056** | 0.102 | 0.429 | 0.388 | 0.020 | 0.429 | 0.470 | 0.2424 |
| **ATGC067** | 0.061 | 0.768 | 0.399 | 0.000 | 0.492 | 0.276 | 0.2243 |
| **ATGC068** | 0.534 | 0.702 | 0.787 | 0.000 | 0.871 | 0.590 | 0.2243 |
| **ATGC071** | 0.088 | 0.149 | 0.316 | 0.009 | 0.843 | 0.702 | 0.2243 |
| **ATGC081** | 0.067 | 0.534 | 0.317 | 0.000 | 0.734 | 0.334 | 0.2243 |
| **ATGC088** | 0.037 | 0.097 | 0.254 | 0.000 | 0.336 | 0.396 | 0.2243 |
| **ATGC089** | 0.083 | 0.131 | 0.220 | 0.028 | 0.818 | 0.667 | 0.2243 |
| **ATGC093** | 0.112 | 1.945 | 0.291 | 0.000 | 1.878 | 0.537 | 0.2243 |
| **ATGC104** | 0.253 | 0.528 | 0.597 | 0.000 | 1.218 | 0.919 | 0.2243 |
| **ATGC105** | 0.000 | 0.892 | 0.248 | 0.050 | 0.941 | 0.693 | 0.2243 |
| **ATGC108** | 0.120 | 0.360 | 0.428 | 0.000 | 0.360 | 0.411 | 0.2424 |
| **ATGC120** | 0.161 | 0.358 | 0.618 | 0.037 | 0.989 | 0.927 | 0.2243 |
| **ATGC127** | 0.036 | 0.193 | 0.290 | 0.000 | 0.941 | 0.627 | 0.2243 |
| **ATGC134** | 0.071 | 0.224 | 0.418 | 0.020 | 0.733 | 0.662 | 0.2243 |
| **ATGC136** | 0.050 | 0.067 | 0.183 | 0.000 | 0.582 | 0.732 | 0.2243 |
| **ATGC137** | 0.069 | 0.529 | 0.806 | 0.000 | 0.967 | 0.713 | 0.2243 |
| **ATGC138** | 0.092 | 0.277 | 0.584 | 0.000 | 0.430 | 0.338 | 0.2243 |
| **ATGC143** | 0.076 | 0.190 | 0.644 | 0.000 | 0.872 | 0.569 | 0.2243 |
| **ATGC144** | 0.000 | 0.000 | 0.208 | 0.000 | 0.052 | 2.185 | 0.2627 |
| **ATGC149** | 0.062 | 0.217 | 0.325 | 0.015 | 0.403 | 0.542 | 0.2243 |
| **ATGC159** | 0.057 | 0.344 | 0.602 | 0.000 | 0.402 | 0.488 | 0.2243 |
| **ATGC184** | 0.327 | 0.414 | 0.305 | 0.000 | 0.828 | 0.785 | 0.2243 |
| **MEAN** | **0.107** | **0.355** | **0.432** | **0.008** | **0.713** | **0.636** | **NA** |

** Chi-test between normalized values and the mean*

**Table S3.** Genome dynamics in defense systems, including gain, loss, expansion and reduction: (a) total number of events; (b) events relative to the number of COGs and (c) events relative to the number of COGs and genomes. Defense systems with less than 10 genes or less than 10 events are left empty.

| **ATGC** | **Genera** | **LS** | **(a) Events (N)** | | | | | **(b) N / COG** | | | | | **(c) N / COG / Genomes** | | | | |
| --- | --- | --- | --- | --- | --- | --- | --- | --- | --- | --- | --- | --- | --- | --- | --- | --- | --- |
|  |  |  | **A** | **C** | **R** | **T** | **U** | **A** | **C** | **R** | **T** | **U** | **A** | **C** | **R** | **T** | **U** |
| **ATGC149** | **Acinetobacter** | FL |  | 37 | 55 | 114 | 99 |  | 2.6 | 2.6 | 4.4 | 2.8 |  | 0.19 | 0.19 | 0.31 | 0.20 |
| **ATGC014** | **Bacillus** | FL | 125 | 116 | 102 | 435 | 137 | 7.8 | 3.5 | 2.4 | 4.7 | 3.1 | 0.25 | 0.11 | 0.08 | 0.15 | 0.10 |
| **ATGC015** | **Bacillus** | FL |  |  | 108 | 83 | 27 |  |  | 4.2 | 2.9 | 1.3 |  |  | 0.17 | 0.12 | 0.06 |
| **ATGC104** | **Bifidobacterium** | FHA | 22 | 30 | 64 | 93 | 60 | 2.0 | 1.3 | 2.4 | 1.8 | 1.5 | 0.18 | 0.12 | 0.22 | 0.16 | 0.14 |
| **ATGC105** | **Bifidobacterium** | FHA |  | 19 | 0 | 26 | 12 |  | 1.1 |  | 1.3 | 0.9 |  | 0.11 |  | 0.14 | 0.09 |
| **ATGC144** | **Borrelia** | FHA |  |  |  |  | 131 |  |  |  |  | 3.1 |  |  |  |  | 0.28 |
| **ATGC136** | **Brucella-Ochrobactrum** | FHA |  |  |  | 54 | 45 |  |  |  | 1.5 | 1.0 |  |  |  | 0.08 | 0.05 |
| **ATGC088** | **Burkholderia** | FL |  | 28 | 101 | 108 | 99 |  | 2.2 | 3.0 | 2.4 | 1.9 |  | 0.17 | 0.23 | 0.19 | 0.14 |
| **ATGC089** | **Burkholderia** | FL | 26 | 19 | 57 | 260 | 167 | 2.2 | 1.0 | 1.8 | 2.2 | 1.7 | 0.18 | 0.08 | 0.15 | 0.18 | 0.14 |
| **ATGC143** | **Campylobacter** | FHA |  |  | 54 | 59 | 41 |  |  | 3.2 | 2.6 | 2.7 |  |  | 0.29 | 0.23 | 0.25 |
| **ATGC044** | **Candidatus-Rickettsia** | P |  |  | 88 | 378 | 49 |  |  | 7.3 | 4.2 | 2.5 |  |  | 0.18 | 0.10 | 0.06 |
| **ATGC021** | **Chlamydia** | P |  |  |  |  |  |  |  |  |  |  |  |  |  |  |  |
| **ATGC022** | **Chlamydia-Chlamydophila** | P |  |  |  |  |  |  |  |  |  |  |  |  |  |  |  |
| **ATGC081** | **Clostridium** | FL |  | 79 | 44 | 81 | 47 |  | 2.5 | 2.3 | 1.8 | 2.4 |  | 0.25 | 0.23 | 0.18 | 0.24 |
| **ATGC067** | **Corynebacterium** | FL |  | 53 | 26 | 24 |  |  | 2.1 | 2.0 | 1.5 | 0.0 |  | 0.12 | 0.11 | 0.08 |  |
| **ATGC068** | **Corynebacterium** | FL | 48 | 111 | 105 | 131 | 41 | 2.5 | 4.4 | 3.7 | 4.2 | 1.9 | 0.20 | 0.34 | 0.29 | 0.32 | 0.15 |
| **ATGC002** | **Enterobacter-Klebsiella** | FHA | 20 | 63 | 89 | 175 | 143 | 1.6 | 2.2 | 1.9 | 2.5 | 2.1 | 0.15 | ` | 0.18 | 0.22 | 0.19 |
| **ATGC001** | **Enterobacteria** | FHA | 200 | 356 | 865 | 1647 | 1643 | 8.0 | 8.9 | 9.7 | 10.4 | 11.6 | 0.07 | 0.08 | 0.09 | 0.10 | 0.11 |
| **ATGC138** | **Francisella** | FHA |  |  | 50 | 40 | 25 |  |  | 2.6 | 2.9 | 2.3 |  |  | 0.15 | 0.16 | 0.13 |
| **ATGC050** | **Helicobacter** | FHA |  |  | 787 | 135 | 297 |  |  | 10.1 | 6.1 | 11.0 |  |  | 0.20 | 0.12 | 0.22 |
| **ATGC056** | **Lactobacillus** | FL |  | 24 | 35 | 37 | 46 |  | 1.1 | 1.8 | 1.7 | 2.0 |  | 0.11 | 0.18 | 0.17 | 0.20 |
| **ATGC184** | **Legionella** | FHA | 54 | 41 | 26 | 92 | 83 | 3.6 | 2.2 | 1.9 | 2.4 | 2.3 | 0.36 | 0.22 | 0.19 | 0.24 | 0.23 |
| **ATGC108** | **Listeria** | FL |  | 145 | 136 | 104 | 69 |  | 6.9 | 5.5 | 4.9 | 2.9 |  | 0.22 | 0.18 | 0.16 | 0.09 |
| **ATGC024** | **Mycobacterium** | FHA | 38 | 70 | 51 | 316 | 69 | 2.7 | 2.1 | 2.7 | 2.5 | 2.1 | 0.09 | 0.06 | 0.08 | 0.08 | 0.07 |
| **ATGC032** | **Mycoplasma** | FHA |  |  |  |  |  |  |  |  |  |  |  |  |  |  |  |
| **ATGC137** | **Neisseria** | FHA |  | 45 | 95 | 120 | 65 |  | 1.9 | 2.7 | 2.9 | 2.1 |  | 0.11 | 0.15 | 0.16 | 0.12 |
| **ATGC159** | **Propionibacterium** | FL |  | 13 | 25 | 16 | 23 |  | 1.1 | 1.2 | 1.2 | 1.3 |  | 0.10 | 0.11 | 0.11 | 0.12 |
| **ATGC071** | **Pseudomonas** | FL |  | 26 | 76 | 244 | 167 |  | 1.5 | 2.1 | 2.5 | 2.1 |  | 0.13 | 0.18 | 0.21 | 0.17 |
| **ATGC120** | **Shewanella** | FL | 57 | 80 | 170 | 205 | 142 | 4.4 | 2.7 | 3.4 | 2.6 | 1.9 | 0.31 | 0.20 | 0.24 | 0.18 | 0.14 |
| **ATGC052** | **Staphylococcus** | FL |  | 110 | 162 | 134 | 319 |  | 6.5 | 7.0 | 5.6 | 5.0 |  | 0.15 | 0.17 | 0.13 | 0.12 |
| **ATGC003** | **Streptococcus** | FL |  |  | 112 | 90 | 85 |  |  | 3.9 | 2.4 | 4.7 |  |  | 0.18 | 0.11 | 0.22 |
| **ATGC004** | **Streptococcus** | FL |  | 48 | 42 | 94 | 120 |  | 3.2 | 3.2 | 4.1 | 2.9 |  | 0.15 | 0.15 | 0.19 | 0.13 |
| **ATGC005** | **Streptococcus** | FL |  | 19 | 57 | 69 | 69 |  | 1.6 | 2.1 | 2.2 | 2.7 |  | 0.10 | 0.13 | 0.14 | 0.17 |
| **ATGC093** | **Sulfolobus** | FL |  | 310 | 29 | 200 | 50 |  | 3.6 | 2.2 | 2.4 | 2.1 |  | 0.30 | 0.18 | 0.20 | 0.17 |
| **ATGC134** | **Xanthomonas** | FHA |  | 44 | 109 | 155 | 182 |  | 2.0 | 2.7 | 2.2 | 2.8 |  | 0.16 | 0.21 | 0.17 | 0.22 |
| **ATGC127** | **Yersinia** | FHA |  | 23 | 52 | 216 | 129 |  | 1.4 | 2.2 | 2.8 | 2.5 |  | 0.07 | 0.11 | 0.15 | 0.13 |

** LS: Life style; A: abortive infection; R: restriction modification; C: CRISPR; T: toxin/antitoxin; U: unknown; FL: free living; P: intracellular parasite; FHA: facultative host associated*

**Table S4. Comparison of the genome dynamics in defense systems (relative to the dynamics in all genes) between life styles using the Welch Two Sample t-test implemented in R**

| **Type of GDE** | **FL vs FHA** | **FL vs P** | **FHA vs P** |
| --- | --- | --- | --- |
|  | **p-value** | | |
| Gain | 0.1539 | 0.06201 | 0.1185 |
| Loss | 0.1229 | 0.2186 | 0.07436 |
| Expansion | 0.3864 | 0.5983 | 0.3857 |
| Reduction | 0.2365 | 0.5158 | 0.2067 |

** FL: free living; P: intracellular parasite; FHA: facultative host associated*

**Table S5. Comparison of the genome dynamics in defense systems (relative to the dynamics in all genes) between taxa using the Welch Two Sample t-test implemented in R**

| **Type of GDE** | **Actinobacteria vs Firmicutes** | **Actinobacteria vs Proteobacteria** | **Firmicutes vs Proteobacteria** |
| --- | --- | --- | --- |
|  | **p-value** | | |
| Gain | 0.7452 | 0.06858 | 0.05135 |
| Loss | 0.3869 | 0.6053 | 0.7081 |
| Expansion | 0.5738 | 0.8872 | 0.8084 |
| Reduction | 0.7274 | 0.7366 | 0.4507 |

**Table S6.** Relative fluxes in defense systems.

| **ATGC** | **Phylum** | **Lifestyle** | **Flux** (relative gene dynamics >75% = high; <25% = low) | **Expanding** (top 25% of gene gain rates) | **Compressing** (top 25% of gene loss rates) |
| --- | --- | --- | --- | --- | --- |
| **ATGC024** | **Actinobacteria** | FHA | LOW | - | - |
| **ATGC067** | **Actinobacteria** | FL | LOW | - | - |
| **ATGC068** | **Actinobacteria** | FL | HIGH | - | - |
| **ATGC104** | **Actinobacteria** | FHA | MEDIUM | - | - |
| **ATGC105** | **Actinobacteria** | FHA | HIGH | - | - |
| **ATGC159** | **Actinobacteria** | FL | LOW | EXPANDING | - |
| **ATGC021** | **Chlamydiae** | P | LOW | - | - |
| **ATGC022** | **Chlamydiae** | P | LOW | - | - |
| **ATGC093** | **Crenarchaeota** | FL | MEDIUM | - | - |
| **ATGC003** | **Firmicutes** | FL | LOW | EXPANDING | - |
| **ATGC004** | **Firmicutes** | FL | MEDIUM | - | - |
| **ATGC005** | **Firmicutes** | FL | MEDIUM | - | - |
| **ATGC014** | **Firmicutes** | FL | LOW | - | - |
| **ATGC015** | **Firmicutes** | FL | MEDIUM | - | - |
| **ATGC052** | **Firmicutes** | FL | MEDIUM | - | - |
| **ATGC056** | **Firmicutes** | FL | MEDIUM | - | - |
| **ATGC081** | **Firmicutes** | FL | MEDIUM | - | COMPRESSING |
| **ATGC108** | **Firmicutes** | FL | HIGH | - | COMPRESSING |
| **ATGC001** | **Proteobacteria** | FHA | HIGH | - | COMPRESSING |
| **ATGC002** | **Proteobacteria** | FHA | HIGH | - | COMPRESSING |
| **ATGC044** | **Proteobacteria** | P | MEDIUM | - | - |
| **ATGC050** | **Proteobacteria** | FHA | HIGH | EXPANDING | COMPRESSING |
| **ATGC071** | **Proteobacteria** | FL | MEDIUM | - | - |
| **ATGC088** | **Proteobacteria** | FL | LOW | EXPANDING | - |
| **ATGC089** | **Proteobacteria** | FL | MEDIUM | - | - |
| **ATGC120** | **Proteobacteria** | FL | MEDIUM | EXPANDING | - |
| **ATGC127** | **Proteobacteria** | FHA | MEDIUM | - | - |
| **ATGC134** | **Proteobacteria** | FHA | HIGH | - | COMPRESSING |
| **ATGC136** | **Proteobacteria** | FHA | LOW | - | - |
| **ATGC137** | **Proteobacteria** | FHA | MEDIUM | - | - |
| **ATGC138** | **Proteobacteria** | FHA | MEDIUM | - | COMPRESSING |
| **ATGC143** | **Proteobacteria** | FHA | HIGH | - | COMPRESSING |
| **ATGC149** | **Proteobacteria** | FL | MEDIUM | EXPANDING | - |
| **ATGC184** | **Proteobacteria** | FHA | HIGH | EXPANDING | COMPRESSING |
| **ATGC144** | **Spirochaetes** | FHA | MEDIUM | - | - |
| **ATGC032** | **Tenericutes** | FHA | MEDIUM | - | - |

** LS: Life style; FL: free living; P: intracellular parasite; FHA: facultative host associated*

**Table S7.** Description of genes in supplementary figures S1, S2 and S3

| **DS Type** | **Code** | **ATGC.COG** | **Annotation** |
| --- | --- | --- | --- |
| **Figure S1** | | | |
| ABI | a1 | ATGC068.Singlet3559 | pfam07751 |
| ABI | a2 | ATGC068.Singlet3525 | pfam08843 |
| ABI | a3 | ATGC068.Singlet3216 | pfam07751 |
| ABI | a4 | ATGC068.COG2752.nosub | pfam08843 |
| ABI | a5 | ATGC068.COG2498.nosub | pfam07751 |
| ABI | a6 | ATGC068.COG2396.1 | COG4849 |
| ABI | a7 | ATGC068.COG2115.singlet3 | COG4823 |
| ABI | a8 | ATGC068.COG2115.singlet2 | COG4823 |
| ABI | a9 | ATGC068.COG2115.singlet1 | COG4823 |
| ABI | a10 | ATGC068.COG2115.1 | COG4823 |
| ABI | a11 | ATGC068.COG2095.1 | COG1106 |
| ABI | a12 | ATGC068.COG2093.1 | COG4849 |
| ABI | a13 | ATGC068.COG2004.1 | pfam07751 |
| ABI | a14 | ATGC068.COG1998.1 | pfam08843 |
| ABI | a15 | ATGC068.COG1962.1 | COG1106 |
| ABI | a16 | ATGC068.COG1801.1 | COG4861 |
| ABI | a17 | ATGC068.COG1573.1 | COG4823 |
| ABI | a18 | ATGC068.COG1371.1 | COG4823 |
| CRISPR | c1 | ATGC068.COG1435.1 | COG1199 |
| CRISPR | c2 | ATGC068.COG1928.1 | cd09643 |
| CRISPR | c3 | ATGC068.COG1929.1 | cd09720 |
| CRISPR | c4 | ATGC068.COG1930.1 | COG3512 |
| CRISPR | c5 | ATGC068.COG1946.1 | pfam09344 |
| CRISPR | c6 | ATGC068.COG1947.1 | cd09719 |
| CRISPR | c7 | ATGC068.COG2206.1 | COG1203 |
| CRISPR | c8 | ATGC068.COG2207.1 | cd09729 |
| CRISPR | c9 | ATGC068.COG2208.1 | cd09731 |
| CRISPR | c10 | ATGC068.COG2209.1 | cd09645 |
| CRISPR | c11 | ATGC068.COG2210.1 | pfam08798 |
| CRISPR | c12 | ATGC068.COG2211.1 | pfam09707 |
| CRISPR | c13 | ATGC068.COG2460.nosub | cd09645 |
| CRISPR | c14 | ATGC068.COG2461.nosub | cd09670 |
| CRISPR | c15 | ATGC068.COG2462.nosub | cls000721 |
| CRISPR | c16 | ATGC068.COG2463.nosub | cd09727 |
| CRISPR | c17 | ATGC068.COG2464.nosub | COG1203 |
| CRISPR | c18 | ATGC068.COG2465.nosub | pfam09707 |
| RM | r1 | ATGC068.COG0008.1 | pfam12161 |
| RM | r2 | ATGC068.COG0032.1 | COG1061 |
| RM | r3 | ATGC068.COG1093.1 | COG1061 |
| RM | r4 | ATGC068.COG1370.1 | COG1715 |
| RM | r5 | ATGC068.COG1770.1 | COG1061 |
| RM | r6 | ATGC068.COG1839.1 | COG0610 |
| RM | r7 | ATGC068.COG1913.1 | COG2189 |
| RM | r8 | ATGC068.COG2036.1 | COG0610 |
| RM | r9 | ATGC068.COG2150.1 | COG2189 |
| RM | r10 | ATGC068.COG2266.nosub | COG4096 |
| RM | r11 | ATGC068.COG2410.nosub | COG0610 |
| RM | r12 | ATGC068.COG2422.nosub | COG4889 |
| RM | r13 | ATGC068.COG2434.nosub | COG0732 |
| RM | r14 | ATGC068.COG2604.nosub | COG0610 |
| RM | r15 | ATGC068.COG2632.nosub | COG2189 |
| RM | r16 | ATGC068.COG2673.nosub | COG4889 |
| RM | r17 | ATGC068.COG2880.nosub | COG3587 |
| RM | r18 | ATGC068.COG2919.nosub | COG0286 |
| RM | r19 | ATGC068.COG2923.nosub | pfam12161 |
| RM | r20 | ATGC068.COG2924.nosub | COG0732 |
| RM | r21 | ATGC068.Singlet3065 | pfam09019 |
| RM | r22 | ATGC068.Singlet3070 | pfam10593 |
| RM | r23 | ATGC068.Singlet3197 | pfam12161 |
| RM | r24 | ATGC068.Singlet3198 | COG4096 |
| RM | r25 | ATGC068.Singlet3231 | COG4889 |
| RM | r26 | ATGC068.Singlet3479 | pfam10544 |
| RM | r27 | ATGC068.Singlet3480 | COG1061 |
| TA | t1 | ATGC068.COG0277.1 | pfam12728 |
| TA | t2 | ATGC068.COG1399.1 | COG3311 |
| TA | t3 | ATGC068.COG1671.1 | COG3177 |
| TA | t4 | ATGC068.COG1747.1 | pfam12728 |
| TA | t5 | ATGC068.COG1776.1 | COG3093 |
| TA | t6 | ATGC068.COG1818.1 | COG3177 |
| TA | t7 | ATGC068.COG1880.1 | COG1476 |
| TA | t8 | ATGC068.COG1923.1 | COG4710 |
| TA | t9 | ATGC068.COG2031.1 | COG2026 |
| TA | t10 | ATGC068.COG2044.1 | COG3177 |
| TA | t11 | ATGC068.COG2050.1 | COG4710 |
| TA | t12 | ATGC068.COG2113.1 | pfam06114 |
| TA | t13 | ATGC068.COG2118.1 | COG2856 |
| TA | t14 | ATGC068.COG2146.1 | pfam13470 |
| TA | t15 | ATGC068.COG2235.1 | pfam12728 |
| TA | t16 | ATGC068.COG2363.1 | COG1476 |
| TA | t17 | ATGC068.COG2500.nosub | COG2184 |
| TA | t18 | ATGC068.COG2566.nosub | COG3654 |
| TA | t19 | ATGC068.COG2596.nosub | COG3177 |
| TA | t20 | ATGC068.COG2601.nosub | COG3550 |
| TA | t21 | ATGC068.COG2602.nosub | pfam05534 |
| TA | t22 | ATGC068.COG2711.nosub | COG3177 |
| TA | t23 | ATGC068.COG2823.nosub | COG4710 |
| TA | t24 | ATGC068.COG2860.nosub | COG4710 |
| TA | t25 | ATGC068.COG2890.nosub | pfam12728 |
| TA | t26 | ATGC068.Singlet3116 | pfam12728 |
| TA | t27 | ATGC068.Singlet3441 | pfam12728 |
| TA | t28 | ATGC068.Singlet3478 | COG3177 |
| TA | t29 | ATGC068.Singlet3527 | COG3549 |
| **Figure S2** | | | |
| ABI | a1 | ATGC081.COG4600.nosub | pfam10592 |
| ABI | a2 | ATGC081.Singlet4818 | pfam10592 |
| CRISPR | c1 | ATGC081.COG0054.1 | COG1199 |
| CRISPR | c2 | ATGC081.COG2537.1 | cd09652 |
| CRISPR | c3 | ATGC081.COG3062.1 | COG1336 |
| CRISPR | c4 | ATGC081.COG3138.1 | cd09679 |
| CRISPR | c5 | ATGC081.COG3603.1 | cd09661 |
| CRISPR | c6 | ATGC081.COG3605.1 | cd09748 |
| CRISPR | c7 | ATGC081.COG3877.nosub | pfam09700 |
| CRISPR | c8 | ATGC081.COG3903.nosub | cd09661 |
| CRISPR | c9 | ATGC081.COG3939.nosub | cd09749 |
| CRISPR | c10 | ATGC081.COG4015.nosub | cd09749 |
| CRISPR | c11 | ATGC081.COG4016.nosub | COG1367 |
| CRISPR | c12 | ATGC081.COG4289.nosub | cd09692 |
| CRISPR | c13 | ATGC081.Singlet5369 | cd09680 |
| CRISPR | c14 | ATGC081.Singlet5396 | cd09683 |
| CRISPR | c15 | ATGC081.Singlet5694 | pfam09701 |
| CRISPR | c16 | ATGC081.Singlet5695 | cd09657 |
| RM | r1 | ATGC081.COG3128.1 | COG1061 |
| RM | r2 | ATGC081.COG3176.1 | pfam12161 |
| RM | r3 | ATGC081.COG3177.1 | COG0732 |
| RM | r4 | ATGC081.COG3178.1 | COG4096 |
| RM | r5 | ATGC081.COG3708.nosub | pfam10544 |
| RM | r6 | ATGC081.COG3899.nosub | COG1061 |
| RM | r7 | ATGC081.COG4047.nosub | pfam10593 |
| RM | r8 | ATGC081.COG4117.nosub | COG4748 |
| RM | r9 | ATGC081.COG4119.nosub | COG0732 |
| RM | r10 | ATGC081.COG4599.nosub | COG0270 |
| RM | r11 | ATGC081.COG4629.nosub | COG0270 |
| RM | r12 | ATGC081.Singlet4813 | COG0610 |
| RM | r13 | ATGC081.Singlet4814 | pfam12161 |
| RM | r14 | ATGC081.Singlet4830 | COG0338 |
| RM | r15 | ATGC081.Singlet5068 | COG0610 |
| RM | r16 | ATGC081.Singlet5083 | COG3183 |
| RM | r17 | ATGC081.Singlet5163 | pfam12161 |
| RM | r18 | ATGC081.Singlet5678 | COG1743 |
| RM | r19 | ATGC081.Singlet5939 | COG0338 |
| TA | t1 | ATGC081.COG0577.1 | COG2337 |
| TA | t2 | ATGC081.COG0677.1 | COG2856 |
| TA | t3 | ATGC081.COG0848.1 | COG1393 |
| TA | t4 | ATGC081.COG0971.1 | COG1476 |
| TA | t5 | ATGC081.COG1466.1 | pfam12728 |
| TA | t6 | ATGC081.COG1537.1 | COG2002 |
| TA | t7 | ATGC081.COG1547.1 | COG1476 |
| TA | t8 | ATGC081.COG1997.1 | COG1476 |
| TA | t9 | ATGC081.COG2274.1 | COG2337 |
| TA | t10 | ATGC081.COG2275.1 | COG0864 |
| TA | t11 | ATGC081.COG2403.1 | COG2002 |
| TA | t12 | ATGC081.COG2505.1 | COG1476 |
| TA | t13 | ATGC081.COG2867.1 | pfam01909 |
| TA | t14 | ATGC081.COG2914. | COG1476 |
| TA | t15 | ATGC081.COG2992.1 | pfam04014 |
| TA | t16 | ATGC081.COG3063.1 | COG1476 |
| TA | t17 | ATGC081.COG3168.1 | COG1476 |
| TA | t18 | ATGC081.COG3294.1 | COG3177 |
| TA | t19 | ATGC081.COG3321.1 | COG2856 |
| TA | t20 | ATGC081.COG3365.1 | COG1476 |
| TA | t21 | ATGC081.COG3781.nosub | COG3654 |
| TA | t22 | ATGC081.COG3833.nosub | COG1476 |
| TA | t23 | ATGC081.COG4393.nosub | pfam12728 |
| TA | t24 | ATGC081.COG4459.nosu | COG2002 |
| TA | t25 | ATGC081.COG4494.nosub | COG1476 |
| TA | t26 | ATGC081.COG4498.nosub | COG3654 |
| TA | t27 | ATGC081.COG4590.nosub | COG1724 |
| TA | t28 | ATGC081.Singlet4736 | COG1476 |
| TA | t29 | ATGC081.Singlet4825 | COG1476 |
| TA | t30 | ATGC081.Singlet4840 | COG1476 |
| TA | t31 | ATGC081.Singlet4846 | COG1724 |
| TA | t32 | ATGC081.Singlet4847 | pfam03681 |
| TA | t33 | ATGC081.Singlet5006 | COG2002 |
| TA | t34 | ATGC081.Singlet5010 | COG1476 |
| TA | t35 | ATGC081.Singlet5010 | COG1476 |
| TA | t36 | ATGC081.Singlet5108 | pfam12728 |
| TA | t37 | ATGC081.Singlet5263 | COG1476 |
| TA | t38 | ATGC081.Singlet5612 | pfam06114 |
| TA | t39 | ATGC081.Singlet5644 | COG2856 |
| TA | t40 | ATGC081.Singlet5708 | COG2856 |
| TA | t41 | ATGC081.Singlet5781 | COG1476 |
| TA | t42 | ATGC081.Singlet5822 | COG1476 |
| TA | t43 | ATGC081.Singlet5840 | COG1476 |
| TA | t44 | ATGC081.Singlet5949 | COG2856 |
| **Figure S3** | | | |
| ABI | a1 | ATGC159.COG2431.nosub | pfam07751 |
| ABI | a2 | ATGC159.COG2383.nosub | COG4823 |
| CRISPR | c1 | ATGC159.Singlet3297 | COG0640 |
| CRISPR | c2 | ATGC159.COG0695.1 | COG1199 |
| CRISPR | c3 | ATGC159.COG2588.nosub | cd09719 |
| CRISPR | c4 | ATGC159.COG2587.nosub | pfam08798 |
| CRISPR | c5 | ATGC159.COG2585.nosub | pfam09344 |
| CRISPR | c6 | ATGC159.COG2584.nosub | cd09731 |
| CRISPR | c7 | ATGC159.COG2583.nosub | cd09729 |
| CRISPR | c8 | ATGC159.COG2582.nosub | cd09641 |
| CRISPR | c9 | ATGC159.COG2140.1 | pfam09707 |
| CRISPR | c10 | ATGC159.COG1956.1 | cd09719 |
| RM | r1 | ATGC159.Singlet3411 | COG1715 |
| RM | r2 | ATGC159.COG2549 | COG1061 |
| RM | r3 | ATGC159.Singlet3316 | COG4889 |
| RM | r4 | ATGC159.Singlet3315 | COG4889 |
| RM | r5 | ATGC159.Singlet3308 | COG0610 |
| RM | r6 | ATGC159.Singlet3305 | COG4889 |
| RM | r7 | ATGC159.Singlet3236 | COG2852 |
| RM | r8 | ATGC159.Singlet2927 | COG4748 |
| RM | r9 | ATGC159.Singlet2926 | COG4748 |
| RM | r10 | ATGC159.GSE2688 | pfam12161 |
| RM | r11 | ATGC159.COG2548.nosub | COG2189 |
| RM | r12 | ATGC159.COG0602.1 | COG1061 |
| RM | r13 | ATGC159.COG0354.1 | COG1061 |
| RM | r14 | ATGC159.COG0119.1 | COG2852 |
| RM | r15 | ATGC159.COG0005.singlet4 | COG2852 |
| RM | r16 | ATGC159.COG0005.singlet3 | COG2852 |
| RM | r17 | ATGC159.COG0005.singlet1 | COG2852 |
| RM | r18 | ATGC159.COG0005.1 | COG2852 |
| TA | t1 | ATGC159.Singlet3398 | COG3177 |
| TA | t2 | ATGC159.Singlet3326 | COG1476 |
| TA | t3 | ATGC159.Singlet3306 | pfam02604 |
| TA | t4 | ATGC159.Singlet3269 | pfam12728 |
| TA | t5 | ATGC159.Singlet3199 | COG3654 |
| TA | t6 | ATGC159.Singlet3197 | COG2856 |
| TA | t7 | ATGC159.Singlet3021 | COG3514 |
| TA | t8 | ATGC159.Singlet2987 | COG2026 |
| TA | t9 | ATGC159.Singlet2986 | xls000612 |
| TA | t10 | ATGC159.Singlet2956 | COG2184 |
| TA | t11 | ATGC159.COG1928.1 | COG4710 |
| TA | t12 | ATGC159.COG1804.1 | COG4691 |
| TA | t13 | ATGC159.COG1750.1 | pfam05534 |
| TA | t14 | ATGC159.COG1615.1 | pfam06114 |

**Table S8.** Locus description of supplementary figure S9

| **GI** | **ATGC_COG** | **COG, pfam or CDC** | **Function** |
| --- | --- | --- | --- |
| **226949361** | ATGC081.COG1442 | COG2108 | Radical SAM superfamily |
| **226949362** | ATGC081.COG3446 |  | hypothetical protein |
| **226949363** | ATGC081.COG3447 |  | hypothetical protein |
| **226949364** | Singlet4808 |  | Serine Recombinase family, fragment |
| **226949365** | Singlet4809 |  | hypothetical protein |
| **226949366** | Singlet4810 |  | N-acetylmuramoyl-L-alanine amidase |
| **226949367** | ATGC081.COG3309 | pfam05105 | Phage holin |
| **226949368** | Singlet4811 |  | hypothetical protein |
| **226949369** | Singlet4812 |  | hypothetical protein |
| **226949370** | ATGC081.COG0145 | COG4443 | ssDNA binding protein PC4 |
| **226949371** | Singlet4813 | COG0610 | HsdR Res subunit |
| **226949372** | ATGC081.COG4119 | COG0732 | Type I restriction modification DNA specificity domain |
| **226949373** | Singlet4814 | COG0286 | type I restriction endonuclease subunit M |
| **226949374** | Singlet4815 |  | hypothetical protein |
| **226949375** | Singlet4816 | COG1204 | Helicase |
| **226949376** | Singlet4817 |  | HEPN domain containing protein, yhm subfamily |
| **226949377** | Singlet4818 |  | AIPR |
| **226949378** | ATGC081.COG4120 | COG1961 | Serine Recombinase (SR) family |
| **226949379** | ATGC081.COG1443 | COG0451 | UDP-N-acetylglucosamine 4-epimerase |

**Table S9. Locus description of supplementary figure S12**

| **GI** | **ATGC_COG** | **COG, pfam or CDC** | **Function** |
| --- | --- | --- | --- |
| **407935118** |  | COG2267 | Lysophospholipase (disrupted) |
| **407935119** | ATGC159.GSE2674 | pfam13340 | hypothetical protein |
| **407935120** | ATGC159.GSE2675 | pfam13586 | transposase for insertion sequence element |
| **407935121** | ATGC159.Singlet2939 | - | single-stranded DNA-binding protein |
| **407935122** | ATGC159.COG2417 | COG1192 | CobQ/CobB/MinD/ParA nucleotide binding domain-containing protein |
| **407935123** | ATGC159.COG2323 | - | hypothetical protein |
| **407935124** | ATGC159.Singlet2940 | COG1430 | hypothetical protein |
| **407935125** | ATGC159.Singlet2941 | - | hypothetical protein |
| **407935126** | ATGC159.Singlet2942 | - | hypothetical protein |
| **407935127** | ATGC159.Singlet2943 | COG3464 | transposase |
| **407935128** | ATGC159.Singlet2944 | - | hypothetical protein |
| **407935129** | ATGC159.Singlet2945 | - | hypothetical protein |
| **407935130** | ATGC159.Singlet2946 | - | hypothetical protein |
| **407935131** | ATGC159.Singlet2947 | COG0798 | Arsenite efflux pump ACR3 |
| **407935132** | ATGC159.Singlet2948 | COG1249 | pyridine nucleotide-disulfide oxidoreductase Lpd |
| **407935133** | ATGC159.COG2607 | cd00090 | arsR family transcriptional regulator |
| **407935134** | ATGC159.COG2608 | pfam06953 | arsenic resistance operon repressor ArsD |
| **407935135** | ATGC159.COG2609 | pfam02374,cd02035 | arsenite-activated ATPase ArsA |
| **407935136** | ATGC159.Singlet2949 | pfam01695 | IstB domain-containing protein ATP-binding protein |
| **407935137** | ATGC159.Singlet2950 | - | hypothetical protein |
| **407935138** | ATGC159.Singlet2951 | - | hypothetical protein |
| **407935139** | ATGC159.Singlet2952 | - | hypothetical protein |
| **407935140** | ATGC159.Singlet2953 | COG4300 | cadmium resistance transporter CadD |
| **407935141** | ATGC159.Singlet2954 | cd00090 | regulatory protein ArsR |
| **407935142** | ATGC159.GSE2681 | cd03768 | resolvase |
| **407935143** | ATGC159.Singlet2955 | cd11586 | VbhA antitoxin to VbhT toxin |
| **407935144** | ATGC159.Singlet2956 | COG2184 | Fic/DOC family |
| **407935145** | ATGC159.Singlet2957 | - | hypothetical protein |
| **407935146** | ATGC159.Singlet2958 | - | hypothetical protein |
| **407935147** | ATGC159.GSE2681 | cd03768,cd00569 | resolvase |
| **407935148** | ATGC159.GSE2674 | pfam13340 | Putative transposase of IS4/5 family |
| **407935149** | ATGC159.GSE2675 | pfam13586 | transposase for insertion sequence element |
| **407935150** |  | COG2267 | Lysophospholipase (disrupted) |

**Table S10. P-values of supplementary figure S14**

| **Genomes** | **DS (by genes)** | **DS (by directons)** | **GL (by genes)** | **GL (by directons)** |
| --- | --- | --- | --- | --- |
| **NC_002935** | <0.001 | 0.008 | 0.197 | 0.148 |
| **NC_016783** | <0.001 | 0.219 | 0.441 | 0.269 |
| **NC_016785** | <0.001 | <0.001 | 0.347 | 0.129 |
| **NC_016787** | <0.001 | 0.373 | 0.339 | 0.41 |
| **NC_016788** | <0.001 | 0.425 | 0.087 | 0.074 |
| **NC_016789** | <0.001 | 0.01 | <0.001 | 0.047 |
| **NC_016790** | 0.002 | 0.452 | 0.003 | 0.154 |
| **NC_016799** | <0.001 | 0.09 | 0.024 | 0.103 |
| **NC_016800** | <0.001 | 0.31 | <0.001 | 0.265 |
| **NC_016801** | <0.001 | 0.049 | <0.001 | 0.53 |
| **NC_016802** | <0.001 | <0.001 | <0.001 | 0.147 |
| **NC_010516** | <0.001 | 0.426 | 0.075 | 0.017 |
| **NC_010520** | <0.001 | 0.851 | <0.001 | 0.111 |
| **NC_012563** | <0.001 | 0.001 | 0.001 | 0.003 |
| **NC_012658** | <0.001 | 0.126 | 0.436 | 0.196 |
| **NC_017297** | 0.009 | 0.256 | 0.39 | 0.37 |
| **NC_017299** | <0.001 | 0.148 | 0.072 | 0.034 |
| **NC_021064** | <0.001 | 0.599 | 0.171 | 0.343 |

** DS = Defense systems; GL = Gains and losses*

**REFERENCES**

1. Novichkov PS, Ratnere I, Wolf YI, Koonin EV, Dubchak I: **ATGC: a database of orthologous genes from closely related prokaryotic genomes and a research platform for microevolution of prokaryotes**. *Nucleic Acids Res* 2009, **37**(Database issue):D448-454.
